# Plasma FGF21 concentrations are regulated by glucose independently of insulin and GLP-1 in lean, healthy humans

**DOI:** 10.7717/peerj.12755

**Published:** 2022-01-19

**Authors:** Thomas P.J. Solomon, Steven Carter, Jacob M. Haus, Kristian Karstoft, Stephanie von Holstein-Rathlou, Mette S. Nielsen, Matthew P. Gillum

**Affiliations:** 1 School of Sport, Exercise and Rehabilitation Sciences, College of Life & Environmental Sciences, University of Birmingham, Edgbaston, United Kingdom; 2 Institute of Metabolism and Systems Research, College of Medical & Dental Sciences, University of Birmingham, Edgbaston, United Kingdom; 3 School of Kinesiology, University of Michigan - Ann Arbor, Michigan, United States of America; 4 Centre of Inflammation and Metabolism, Rigshospitalet, Copenhagen, Denmark; 5 Centre for Physical Activity Research, Rigshospitalet, Copenhagen, Denmark; 6 Novo Nordisk Foundation Center for Basic Metabolic Research, Faculty of Health and Medical Sciences, University of Copenhagen, Copenhagen, Denmark

**Keywords:** Fibroblast growth factor 21, Secretagogue, Incretin hormones, Clamp methodology, FGF21 secretion

## Abstract

**Background:**

Fibroblast growth factor 21 (FGF21) treatment improves metabolic homeostasis in diverse species, including humans. Physiologically, plasma FGF21 levels increase modestly after glucose ingestion, but it is unclear whether this is mediated by glucose itself or due to a secondary effect of postprandial endocrine responses. A refined understanding of the mechanisms that control FGF21 release in humans may accelerate the development of small-molecule FGF21 secretagogues to treat metabolic disease. This study aimed to determine whether FGF21 secretion is stimulated by elevations in plasma glucose, insulin, or glucagon-like peptide-1 (GLP-1) in humans.

**Methods:**

Three groups of ten healthy participants were included in a parallel-group observational study. Group A underwent a hyperglycemic infusion; Group B underwent a 40 mU/m^2^/min hyperinsulinemic euglycemic clamp; Group C underwent two pancreatic clamps (to suppress endogenous insulin secretion) with euglycemic and hyperglycemic stages with an infusion of either saline or 0.5 pmol/kg/min GLP-1. Plasma FGF21 concentrations were measured at baseline and during each clamp stage by ELISA.

**Results:**

Plasma FGF21 was unaltered during hyperglycemic infusion and hyperinsulinemic euglycemic clamps, compared to baseline. FGF21 was, however, increased by hyperglycemia under pancreatic clamp conditions (*P* < 0.05), while GLP-1 infusion under pancreatic clamp conditions did not change circulating FGF21 levels.

**Conclusion:**

Increases in plasma FGF21 are *likely* driven directly by changes in plasma glucose independent of changes in insulin or GLP-1 secretion. Ecologically valid postprandial investigations are now needed to confirm our observations from basic science infusion models.

## Introduction

Fibroblast growth factor 21 (FGF21) is a liver-derived hormone that regulates nutrient homeostasis in a species-specific manner (Kharitonenkov et al., 2005; Fisher & Maratos-Flier, 2016). Pharmacological doses of FGF21 normalize blood glucose and triglyceride levels in *ob/ob* and *db/db* mice and ameliorate obesity and metabolic dysfunction in murine models of high fat diet-induced obesity as well as in obese and diabetic primates (Kharitonenkov et al., 2005; Xu et al., 2009; Adams et al., 2013; Andersen et al., 2018; Wang et al., 2019)*.* In humans with type 2 diabetes, FGF21 analogs also improve lipids and markers of insulin sensitivity, but without lowering plasma glucose levels (Gaich et al., 2013; Talukdar et al., 2016b), whereas studies investigating the weight-lowering effect of FGF21 in humans report variable outcomes (Gaich et al., 2013; Talukdar et al., 2016b; Kim et al., 2017; Charles et al., 2019). Despite the therapeutic efficacy of FGF21 in humans, knowledge of both its physiological functions and regulation *in vivo* remains incomplete.

In rodents, FGF21 production is triggered by the lipid-activated transcription factor peroxisome proliferator-activated receptor alpha (PPARα) to promote triglyceride catabolism and ketosis during fasting and ketogenic diet feeding (Badman et al., 2007). Interestingly, increased FGF21 in these contexts may also be due to a stimulatory effect of protein-restriction on FGF21 (Laeger et al., 2014), possibly through activating transcription factor 4 (ATF4), caused by starvation or the extremely high-fat ketogenic diets, suggesting that its secretion may be sensitive to the abundance—and/or relative abundance—of multiple nutrients in the liver. In further support of this concept, sugars also increase circulating FGF21 levels by activating the nuclear transcription factor carbohydrate response element-binding protein (ChREBP) in the liver (Von Holstein-Rathlou et al., 2016). Thus, FGF21 production is induced by fatty acids and saccharides but repressed by amino acids in mice, and seems to be increased to the greatest extent by low-protein, high-carbohydrate diets (Solon-Biet et al., 2016). In addition to being regulated by nutrient-responsive transcription factors, FGF21 production is influenced by circulating factors including glucagon, growth hormone, glucocorticoids, and leptin (Erickson & Moreau, 2017). Yet, why FGF21 fluctuates in response to different nutrients, nutrient combinations, and circulating factors is not completely understood. We, and others, found that FGF21 suppresses sugar appetite without dampening hunger for complex carbohydrates, proteins, or lipids in mice (Talukdar et al., 2016a; Von Holstein-Rathlou et al., 2016). Based on these observations, we proposed FGF21 to be the centerpiece of a physiological saccharide-triggered ChREBP-dependent cascade that limits sugar appetite to promote intake of other nutrients once a certain level of carbohydrate consumption is achieved. Evidence from association studies indicates that such a pathway also exists in humans (Søberg et al., 2017; Frayling et al., 2018; Meddens et al., 2020), but randomized controlled trials are needed to assess causality. For therapeutic approaches to target such a pathway and limit palatable nutrient intake, more knowledge will be needed on the mechanisms—ChREBP or otherwise—that stimulate FGF21 production in humans.

In humans, oral boluses of sucrose, glucose, and fructose elevate circulating FGF21 levels two-fold within two hours (Lin et al., 2012; Dushay et al., 2015; Søberg et al., 2017; Samms et al., 2017). Furthermore, carbohydrate overfeeding increases FGF21 levels 8-fold when protein intake is held constant whereas fat overfeeding does not increase FGF21 (Lundsgaard et al., 2017). And, in contrast to rodents, at least seven days of starvation are required for FGF21 levels to rise modestly (2-3 fold) (Gälman et al., 2008; Fazeli et al., 2015), arguing against protein restriction or increased fatty acid delivery to the liver as major mechanisms for FGF21 production in humans. Finally, clinical studies that have directly examined the effect of protein depletion on FGF21 production have reported up to 6-fold increases but are complicated by the fact that these subjects, in addition to eating less protein, also consumed more energy and carbohydrate overall to maintain caloric balance (Laeger et al., 2014; Maida et al., 2016). However, a recent study investigated the induction of FGF21 production following 24 h of 7 dietary interventions with different macronutrient content. Plasma FGF21 increased three fold only after the low-protein/high-carbohydrate and the low-protein/high-fat diets (Vinales et al., 2019). Thus, current evidence suggests that FGF21 production in humans is primarily determined by carbohydrate intake, with more work needed to understand the regulatory contribution of protein, as well as the ingested carbohydrate to protein ratio.

In terms of mechanisms, understanding of FGF21 secretion in humans is limited; however, there is evidence that both the insulin to glucagon ratio and the direct activation of hepatic ChREBP by sugars may play a role in stimulating FGF21 secretion (Hansen et al., 2015; Fisher et al., 2017). While it is clear that carbohydrates increase plasma FGF21 levels in humans, it is not known whether postprandial secretion of FGF21 in response to carbohydrate is evoked directly by nutrients or whether it is secondary to an endocrine response to nutrient ingestion (*e.g.*, insulin, incretins, or other hormone secretion). A study in individuals with obesity has suggested that increased insulin but not glucose is responsible for postprandial increases in FGF21 (Samms et al., 2017). However, since FGF21 metabolism is altered in obesity, we still lack clear knowledge in lean healthy cohorts. Since FGF21 release from the liver accounts for changes in circulating levels under normal conditions (Markan et al., 2014; Hansen et al., 2015), and since saccharides can directly stimulate FGF21 secretion *in vitro via* ChREBP, we hypothesized that glucose-induced changes in circulating concentrations of FGF21 in humans occur independently of variation in insulin or incretin hormones. Therefore, we set out to determine whether plasma FGF21 levels in lean, healthy individuals are influenced by glucose, insulin, or GLP-1 using glucose/insulin infusions and pancreatic clamp methodology.

## Materials & Methods

### Participants

Three independent groups of participants were recruited for three independent studies whereby individuals underwent a hyperglycemic infusion (*n* = 10 healthy male participants, age 23 ± 1 years and BMI 22.7 ± 0.8 kg/m^2^), a hyperinsulinemic euglycemic clamp (*n* = 10 healthy participants, 4 male, 6 female, age 27 ± 1 years and BMI 22.3 ± 0.8 kg/m^2^), or a pancreatic clamp (*n* = 10 healthy male participants, age 22 ± 1 years and BMI 21.2 ± 0.5 kg/m^2^). Some subject data from these studies have been previously published (Karstoft et al., 2015; Mahmoud et al., 2016; Carter & Solomon, 2020). Potential participants provided written informed consent before participation, and were screened to assess their eligibility to partake. Individuals were excluded if they had any evidence of chronic disease. Subject characteristics are shown in Table 1. All procedures were performed per the declaration of Helsinki. Trials were registered at clinicaltrials.gov (NCT03284216, NCT01749163, NCT03284216) and approved by the following research ethics committees: The Ethical Committee of the Capital Region of Denmark, the Institutional Review Boards of the Cleveland Clinic and the University of Illinois at Chicago.

**Table 1:** Subject characteristics.

Subject characteristics	Hyperglycemic clamp	Hyperinsulinemic euglycemic clamp	Pancreatic clamp
N (M/F)	10 (10)	10 (4/6)	10 (10/0)
Age (years)	23 ± 1	27 ± 1	22 ± 1
Weight (kg)	69.9 ± 2.8	64.3 ± 3.9	70.0 ± 2.1
BMI (kg/m^2^)	22.7 ± 0.8	22.3 ± 0.8	21.2 ± 0.5
HbA1c (%)	5.3 ± 0.1	5.3 ± 0.1	5.3 ± 0.1
HbA1c (mmol/mol)	34.8 ± 1.2	34.0 ± 0.8	34.2 ± 0.7

**Notes.**

Data are presented as mean ± SEM. One-way ANOVA was used to compare subject characteristics between groups. There were no significant differences between groups.

### Pre-trial standardization

Prior to the trials in the hyperglycemic clamp and hyperinsulinemic clamp studies, participants were instructed to continue their normal diet and avoid vigorous physical activity and alcohol for 48 h prior to the trials. In the pancreatic clamp study, participants were instructed to avoid vigorous physical activity and alcohol for 48 h prior to the trials; and, diet records were taken for 24 h prior to the first trial, and subjects were instructed to ingest the same foods prior to the subsequent trial.

### The hyperglycemic infusion study

To determine the effect of experimentally-elevated plasma glucose on plasma FGF21 levels, participants arrived in the lab following an overnight fast and underwent a continuous constant-rate glucose infusion to establish a steady hyperglycaemic profile. Specifically, 1.2 g/kg glucose was infused at a constant infusion rate across 3.5 h. Arterialized venous blood glucose was measured at the bedside every 5 min throughout (Hemocue; Radiometer, Copenhagen, Denmark). Venous blood samples were collected into EDTA-containing blood tubes at baseline and after 3.5-hours of hyperglycemia. Plasma was separated by centrifugation and stored at −80 °C before analysis for FGF21.

### The hyperinsulinemic euglycemic clamp study

To determine the effect of experimentally-elevated plasma insulin concentrations on plasma FGF21 levels, participants came to the lab following an overnight fast and underwent a 2-hour hyperinsulinemic euglycemic clamp. Full methodological details are available elsewhere (Mahmoud et al., 2016). In brief, a primed, constant 40 mU/m^2^/min intravenous infusion of insulin (Novolin, Novo Nordisk, Plainsboro, NJ) was administrated. To “clamp” plasma glucose levels at fasting levels (5 mM), exogenous dextrose monohydrate was simultaneously infused at a variable rate according to a computed algorithm. Arterialized venous blood glucose was measured every 5 min (YSI 2300 Stat Plus, Yellow Springs, OH, USA). Venous blood samples were collected into EDTA-containing blood tubes at baseline and after 2 h of hyperinsulinemic euglycemia. Plasma was separated by centrifugation and stored at −80 °C before analysis for FGF21.

### The pancreatic clamp study

To determine the effect of experimental elevation of plasma glucose levels on plasma FGF21 concentrations independent of changes in insulin secretion, participants arrived in the lab following an overnight fast and underwent a pancreatic clamp. Full methodological descriptions are available elsewhere (Karstoft et al., 2015). Briefly, to prevent endogenous pancreatic endocrine activity, somatostatin (Octreotide, Hospira, Lake Forest, IL) was infused at 100 ng/kg/min. Actrapid (0.15 mU/kg/min; Novo Nordisk, Bagsvaerd, Denmark), GlucaGen (0.5 ng/kg/min; Novo Nordisk), and Humatroph (3 ng/kg/min; Eli Lilly, Indianapolis, IN, USA) were also infused at constant rates to restore basal plasma levels of insulin, glucagon, and growth hormone, respectively. Pancreatic clamps lasted for 3-hours and began with 90-minutes at euglycemia followed by 90-minutes at hyperglycemia. Plasma glucose was “clamped” at basal levels (euglycemia) or 5.4 mM above basal (hyperglycemia) *via* a variable-rate exogenous dextrose monohydrate infusion, adjusted according to a computed algorithm. The same subjects also underwent a separate pancreatic clamp trial, performed 1 to 2 weeks apart, whereby GLP-1_7−36_ amide (Polypeptide Laboratories, Hillerød, Denmark; dissolved in sterilized water containing 2% wt/vol human serum albumin) was also infused throughout at a rate of 0.5 pmol/kg/min. Arterialized venous blood glucose was measured at the bedside every 5 min (ABL 725; Radiometer, Copenhagen, Denmark). Venous blood samples were collected into EDTA-containing blood tubes at baseline, and after the euglycemic and hyperglycemic stages of the clamp. Plasma was separated by centrifugation and stored at −80 °C before analysis for FGF21.

### Plasma FGF21 analysis

FGF21 concentrations were quantified using a commercially available sandwich ELISA (BioVendor, Kassel, Germany). Our inter- and intra-assay CVs using this assay were 3.82% and 9.18% respectively.

### Statistics

Plasma FGF21 levels from the three independent studies were tested for normality and were deemed to be normally distributed so parametric statistics were used. Independent, two-tailed, paired t-tests were used to examine changes in FGF21 during the hyperglycemic infusion (basal *vs.* hyperglycemia), during the hyperinsulinemic euglycemic clamp (basal *vs.* hyperinsulinemic euglycemia), and during the pancreatic clamp (euglycemia *vs.* hyperglycemia). Two-way repeated-measures ANOVA was used to compare the pancreatic clamps control and GLP-1 trials. Tukey *post hoc* tests were used to adjust for multiple comparisons. Differences in participant characteristics between the three study groups were compared by one-way ANOVA. Statistical analyses were performed with Prism version 8 (GraphPad, La Jolla, CA, USA), and statistical significance was achieved when *P* < 0.05.

## Results

**Figure 1:**
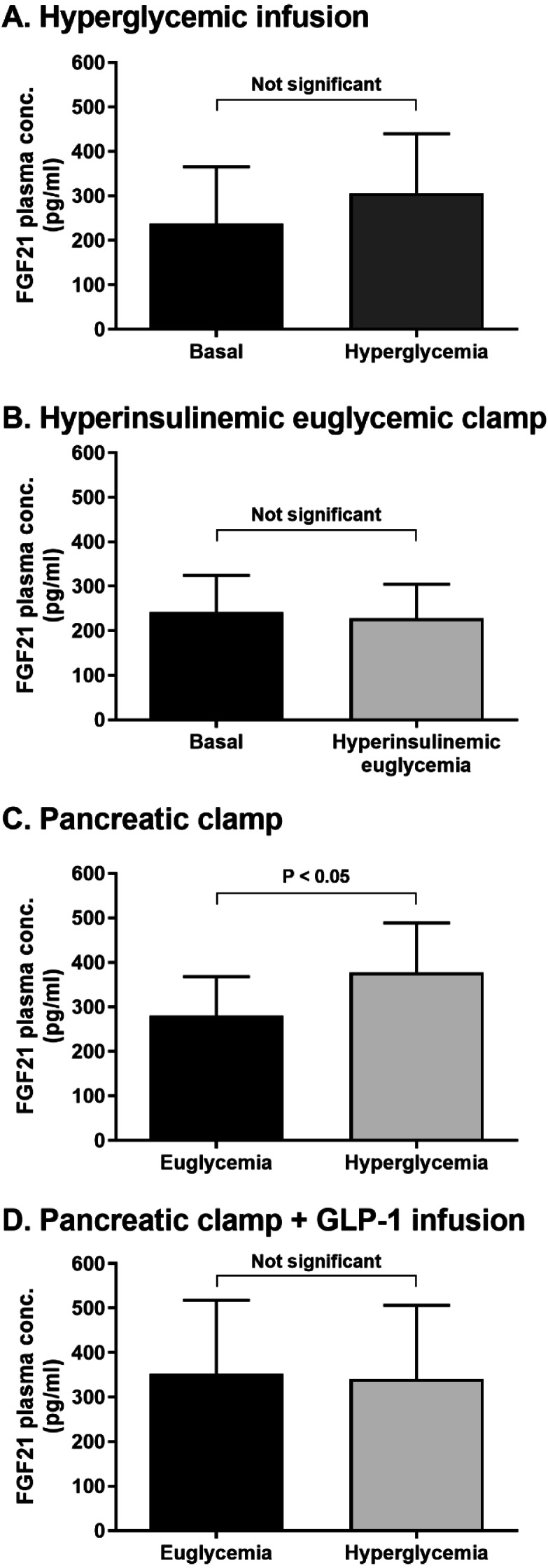
Plasma FGF21 responses to experimental elevations in plasma glucose, plasma insulin, or both. Plasma FGF21 responses were determined by recruiting healthy participants to undergo a hyperglycemic infusion (A), a hyperinsulinemic euglycemic clamp (B), a pancreatic clamp with basal insulin replacement during euglycemic and hyperglycemic stages (C), or a pancreatic clamp with basal insulin replacement during euglycemic and hyperglycemic stages combined with intravenous GLP-1 infusion (D). FGF21 concentrations were measured by ELISA. Independent, paired t-tests were used to compare means in the separate clamp studies. Data are presented as mean ± SEM for ten participants in each study. Due to analytical failure in the FGF21 analysis, 1 subject’s data was lost from the hyperinsulinemic euglycemic clamp data set, leaving *N* = 9 for FGF21 comparisons in B.

Three different groups of ten participants completed the clamps in each study. Participants in each of the three clamp studies had similar age, BMI, and HbA1c levels (Table 1). Clamps were conducted with a high level of quality as reflected by low coefficients of variation in steady-state plasma glucose levels (Table 2). Neither experimental hyperglycemia with the expected rise in plasma insulin levels (Fig. 1A and Table 2: paired *t*-test *P* = 0.10) nor experimental hyperinsulinemia with plasma glucose clamped at euglycemic levels (Fig. 1B and Table 2: paired *t*-test statistic *P* = 0.16), had any effect on plasma FGF21 concentrations when compared to basal levels. However, experimental hyperglycemia (with somatostatin-induced suppression of endogenous insulin secretion and basal insulin replacement) increased plasma FGF21 concentrations above levels measured during euglycemia (Fig. 1C and Table 2: paired *t*-test statistic *P* = 0.01). Meanwhile, during a GLP-1 infusion that significantly increased plasma GLP-1 to 2783 ± 239 and 2500 ± 183 pg/mL from a fasting level of 1348 ± 115 pg/mL (*P* < 0.05), plasma FGF21 concentrations were not different between euglycemia and hyperglycemia (Fig. 1D and Table 2: paired *t*-test statistic *P* > 0.99). During these pancreatic clamp studies, two-way ANOVA revealed a trial-time interaction (*P* < 0.05) and post hoc analyses indicated a significant difference (*P* < 0.05) in FGF21 levels between hyperglycemia and euglycemia in the non-GLP-1 control trial only.

**Table 2:** Clamp characteristics.

Clamp characteristics		Plasma glucose		Plasma insulin	Plasma FGF21
		(mmol/L)	(CV, %)	(pmol/L)	(pg/mL)
Hyperglycemic clamp:					
	0-hours	5.02 ± 0.27	n/a	40.0 ± 4.0	237 ± 128
	3.5-hours	6.94 ± 0.41	1.53 ± 0.26	146 ± 43	306 ± 134
Hyperinsulinemic euglycemic clamp:					
	0-hours	4.76 ± 0.27	n/a	36.4 ± 6.6	242 ± 82
	2-hours	4.96 ± 0.05	6.47 ± 0.83	458 ± 13^*^	228 ± 77
Pancreatic clamp:					
	Euglycemic	5.19 ± 0.20	2.14 ± 0.30	76.9 ± 6.2	299 ± 105
	Hyperglycemic	10.5 ± 0.2^***^	3.22 ± 0.88	95.4 ± 6.3	406 ± 135^*^
Pancreatic clamp + GLP-1:					
	Euglycemic	4.94 ± 0.19	1.34 ± 0.19	85.3 ± 3.1	352 ± 166
	Hyperglycemic	10.3 ± 0.2^***^	3.73 ± 0.63	158 ± 15^**^	341 ± 166

**Notes.**

Data are presented as mean ± SEM for ten participants in each study. Due to analytical failure in the FGF21 analysis, 1 subject’s data was lost from the hyperglycemic infusion and the hyperinsulinemic euglycemic clamp data sets, leaving *N* = 9 for FGF21 comparisons in those studies. Independent, paired t-tests were used to compare time-points for each variable in the separate clamp studies.

* *P* < 0.05.

** *P* < 0.01.

*** *P* < 0.001.

Represents the significant difference versus the previous time-point within the clamp.

## Discussion

These findings show that in lean, healthy individuals, the plasma FGF21 response to an increase in plasma glucose is independent of changes in insulin and GLP-1 secretion. This suggests that glucose *per se* triggers FGF21 secretion in humans. Considering that FGF21 is primarily secreted from the liver (Markan et al., 2014; Hansen et al., 2015), it is prudent to speculate that absorbed glucose directly stimulates hepatic FGF21 secretion.

Even though we did not detect a rise in plasma FGF21 levels following the 3.5-hours experimental hyperglycemia, we previously reported that plasma FGF21 levels increase 3-fold after 24 h of experimental hyperglycemia (Von Holstein-Rathlou et al., 2016). Our finding that FGF21 levels are not increased during hyperinsulinemic euglycemic conditions suggests that FGF21 secretion is unlikely to be regulated by the insulin secretory response to ingested carbohydrates. Instead, it is probable that, as in the rodent (Von Holstein-Rathlou et al., 2016), glucose and its metabolites directly activate ChREBP in the liver to promote FGF21 transcription. This view is supported by recent observations that rates of *de novo* lipogenesis, a process that is coordinated at the transcriptional level by ChREBP, correlate with plasma FGF21 concentrations (Fisher et al., 2017). Similarly, that FGF21 is related to the insulin to glucagon ratio, and that somatostatin-induced blockade of insulin and glucagon responses abolishes exercise-induced increases in FGF21 (Hansen et al., 2015), suggest that the driver of oral glucose-induced FGF21 responses may actually be hormone-driven (*e.g.*, insulin) changes in glycemia, as opposed to the direct actions of the hormones themselves. This conclusion is further consistent with the reports that nutrients like fructose (Dushay et al., 2015) and alcohol (Desai et al., 2017; Søberg et al., 2018; Song et al., 2018), which do not invoke an insulin response, are capable of increasing FGF21 production in humans. While prior work has indicated that GLP-1 analogs or GLP-1 receptor agonists may influence plasma FGF21 levels in rodents (Nonogaki, Hazama & Satoh, 2014; Nonogaki et al., 2016), our work demonstrates that GLP-1 does not directly affect circulating FGF21 levels in humans. Although, it must be noted that, as shown in Table 2, it is difficult to fully isolate the direct effects of GLP-1 since GLP-1 infusion during hyperglycemic portion of the pancreatic clamp slightly increased plasma insulin despite somatostatin infusion. Finally, the lack of data during the clamps is a limitation of this study. All that said, it is likely that efforts to develop small molecules to increase FGF21 for therapeutic applications may be most productively directed at modifying the activity of intracellular nutrient-sensing pathways, as opposed to endocrine signaling pathways.

Although there is now considerable evidence that sugars and other carbohydrates stimulate FGF21 production in humans, an important question not addressed by our work remains. Namely, why it is that plasma FGF21 levels do not increase following ingestion of mixed meals that contain significant amounts of carbohydrates (Umberger et al., 2014; Vienberg et al., 2017)? One explanation is that absorption of glucose from starches (complex carbohydrates) concomitantly mixed with protein and fat is delayed, resulting in lower peak intrahepatic glucose levels and stimulation of carbohydrate-responsive transcriptional programs that elevate FGF21. Another possibility is that other nutrients or factors inhibit FGF21 production in this context. Amino acid deprivation, for example, is known to induce FGF21 production *via* ATF4 (De Sousa-Coelho, Marrero & Haro, 2012). It is therefore conceivable that elevated intrahepatic amino acid levels may antagonize ChREBP-mediated induction of FGF21 transcription. However, this model remains speculative, and limited work has been done to date on factors that inhibit FGF21 production in the presence of stimuli that normally would enhance its secretion.

A broader question that emerges in light of this work is why a system would evolve in this way, to release—in proportion to the amount of carbohydrate consumed—a metabolic hormone from the liver to promote peripheral glucose uptake and lipid catabolism, as well as acting centrally to reduce sugar appetite. A possibility is that the liver, which integrates whole-body energy homeostasis and substrate interconversion, needs to communicate with the central nervous system, which controls energy intake, to regulate both organ and organismal nutrient homeostasis. For example, excess nutrient load in the liver may cause hepatic nutrient stress, leading to pathological outcomes, as is clear from a large number of endoplasmic reticulum stress models (Rutkowski et al., 2008). In addition, the liver is also able to monitor the details of peripheral energy utilization in ways that the brain cannot. For instance, 90% of fructose is absorbed in the hepatic “first pass”, and as such, the calories contained therein cannot be directly sensed by the brain. Thus, it stands to reason that systems might exist to directly sense such nutrients *via* nuclear transcription factors, which allow for stoichiometric production of their targets and according to a graded response proportional to what was ingested.

## Conclusions

Work in individuals with obesity suggests that insulin and not glucose may drive postprandial FGF21 secretion (Samms et al., 2017). Here we show that increases in plasma FGF21 are likely driven directly by changes in plasma glucose independent of changes in insulin or GLP-1 secretion. Whether this holds under ecologically valid postprandial conditions remains to be examined. In addition, insulin-dependent inhibition of lipolysis and proteolysis during the hyperinsulinemic-euglycemic clamp and pancreatic clamp with GLP-1 infusion may also have contributed to the lack of FGF21 induction observed in these conditions by changing the availability of amino acids and fatty acids in the liver. That said, our straightforward approach using gold standard glucose and insulin clamp methodology advances the physiological understanding of FGF21 secretion in lean and healthy humans. Our findings have a clinical impact since gaining mechanistic insight into FGF21 secretion will enhance developments in pharmaceutical targeting of FGF21 signaling and appetite control systems. Physiological postprandial models are now warranted to confirm that ingested glucose directly stimulates FGF21 secretion independent of the postprandial insulin and GLP-1 responses.

## Supplemental Information

10.7717/peerj.12755/supp-1 Supplemental Information 1 Raw data

10.7717/peerj.12755/supp-2 Supplemental Information 2 Protocol for the hyperglycemic infusion study

10.7717/peerj.12755/supp-3 Supplemental Information 3 Protocol for the hyperinsulinemic euglycemic clamp study

10.7717/peerj.12755/supp-4 Supplemental Information 4 Protocol for the pancreatic clamp study

10.7717/peerj.12755/supp-5 Supplemental Information 5 Flow diagram

## Additional Information and Declarations

### Data Availability

The following information was supplied regarding data availability: The raw data are available in the Supplementary File.

### Clinical Trial Registration

The following information was supplied regarding Clinical Trial registration: NCT03284216, NCT01749163, NCT03284216.
