# Supplementary material for: Plasma FGF21 concentrations are regulated by glucose independently of insulin and GLP-1 in lean, healthy humans
Source: PeerJ. 2022 Jan 19;10:e12755. doi: 10.7717/peerj.12755 (PMC8783558; doi:10.7717/peerj.12755)
Supplement: Supplemental Information 2 [file peerj-10-12755-s002.pdf]

Information sheet for prospective volunteers

School of Sport, Exercise & Rehabilitation Sciences
University of Birmingham
Edgbaston
Birmingham B15 2TT
United Kingdom

Telephone:	+44 (0)121 414 4115
Fax:	+44 (0)121 414 4121
Web:	www.birmingham.ac.uk/schools/sport-exercise/index.aspx

The effects of hyperglycaemia on the response to acute exercise

We would like to invite you to take part in a research study. Before you decide we would like you to understand why the research is being done and what it would involve for you. One of our team will go through the information sheet with you and answer any questions you have. This should take about 5 minutes. But please talk to others about the study if you wish. Please also ask us if there is anything that is not clear.

Where does the study take place?

The School of Sport, Exercise and Rehabilitation Sciences at the University of Birmingham.

Who are the study investigators?

Mr Steven Carter (Doctoral Researcher, and Chief Investigator) and Dr Thomas Solomon (Senior Lecturer & EU Marie Curie Research Fellow, and Principle Investigator).

Who is funding the research?

This project is being funded by Dr Thomas Solomon's research budget.

Who has reviewed the study?

The study has been reviewed by the National Research Ethics Service West Midlands Research Ethics Committee (Protocol ID: *ERN_16-0193*; IRAS ID number: *202409*).

Expenses and payments

We will reimburse you up to £50 per experimental trial for your travel expenses and time.

Study background, rationale and purpose:

High blood glucose levels (also described as hyperglycaemia) impair metabolic and endocrine function. Previous research has consistently demonstrated the effectiveness of exercise training in improving the ability to regulate and maintain blood glucose levels within normal ranges (i.e. glycaemic control). However, even within well-controlled studies, the effectiveness of exercise is extremely variable, with evidence demonstrating smaller benefits in individuals with high pre-training blood glucose levels (e.g. diabetic patients). Furthermore, fluctuating blood glucose levels causes greater impairments than stable high glucose levels. However, the precise mechanisms explaining the blunted exercise adaptations and the impact of the pattern of high blood glucose levels upon the benefits of exercise are unknown. Additionally, whether adaptations to long-term physical activity (such as elevated physical fitness) provide protection against the negative effects of hyperglycaemia remains to be established.

Given the aforementioned gaps in existing knowledge, investigating whether high blood glucose levels (of differing patterns) directly blunts improvements in glycaemic control following a single bout of exercise will improve our understanding of the large variability in the beneficial response to exercise seen in individuals with high blood glucose. Such new knowledge may have clinical

relevance since it may aid the development of future optimised and individualised approaches to using exercise for the improvement of glycaemic control in diabetes patients, for example.

Accordingly, the overall aim of this research study is to determine whether exposure to short-term high blood glucose levels impairs the exercise-induced adaptations in glucose tolerance following a single exercise bout in healthy individuals, and whether adaptations to long-term physical activity and/or the pattern of high blood glucose levels influence this response.

Do I have to take part?

No, you are under no obligation to participate in this study. Participation in this study is entirely voluntary. If you would like to participate, you will be given this information sheet to keep and be asked to sign a consent form. You are still free to withdraw at any time up until the morning of the final trial and may do so without giving a reason.

Am I eligible for this study?

You are likely to be eligible for this study if you fulfil the following criteria:

- Male
- Age 18-50 years
- Body Mass Index 19-30 kg/m^2 (weight in kg divided by height in metres, squared)
- Generally healthy
- Non-smoker
- Do not regularly use anti-inflammatory medication
- Have not had more than 2 kg weight change in the last 6 months
- Have not undergone weight loss surgery
- Do not have, or have not had, cancer or chronic haematological, pulmonary, cardiac, hepatic, renal, metabolic (e.g. diabetes) or gastrointestinal diseases

May I be excluded from the study?

Yes. We, as a research team, expect you to adhere to the study conditions. You can be excluded from the study at any time point if you do not adhere to the study conditions.

What are the benefits of taking part in this study?

Benefits for you: We will provide you with feedback on your fitness level ($\dot{V}O_2max$), and measures of your metabolic and cardiovascular health. We will also be happy to discuss how you can use exercise to help improve your overall health.

Wider benefits: In the long-term we hope that the information gained from your participation will be useful to devise optimised and individualised approaches to using exercise for the improvement of glycaemic control in diabetes patients, for example.

What will I have to do?

To complete the study we are looking to recruit individuals to take part in this study between 29th May 2017 and 31st December 2018. It will involve the following five visits to the School of Sport, Exercise and Rehabilitation Sciences at the University of Birmingham. This will consist of one screening visit and four experimental trials.

Visit 1 – Screening Visit (~2 hours). If you decide to participate in the study you will be invited to a screening visit. For this visit, we ask that you come to the School of Sport, Exercise and Rehabilitation Sciences at the University of Birmingham having not eaten or drunk anything except water for 4-hours, as well as having refrained from alcohol consumption and vigorous physical activity in the 48-hours preceding the visit. This will ensure that you are able to perform maximally during the maximal exercise test, as well as to avoid any discomfort or vomiting.

A study investigator will explain the project further and ask if you understand everything in this Participant Information Sheet. You will then have the chance to ask any questions you have. At this point, if you are happy to participate in the study you will be asked to sign an Informed Consent

Form to document that you wish to participate further. You will then be asked to complete a General Health Questionnaire about your health and activity status as well as having your body weight and height measured and body mass index (BMI = weight in kg / height in m^2) calculated, and your body composition measured using skinfold callipers. The questionnaire and weight/height measurements are necessary to determine your eligibility to continue in the study. If you successfully complete the General Health Questionnaire, a blood sample will be collected and we will ask you to perform an exercise test to voluntary exhaustion for us to determine your maximal oxygen uptake ($\dot{V}O_2max$, or fitness level). This test will involve cycling in the lab initially at a low power output of ~50-100 watts. This will be followed by incremental stages at progressively higher workloads, increasing by 15-30 watts every 1-3 minutes. The test will continue until you cannot cycle any further (you reach your maximum). Throughout the test we will monitor your heart rate using a chest-strap and watch, your blood pressure using an automated cuff, and also ask you to breathe into a mouthpiece connected to a computer to calculate your oxygen uptake. The data obtained from this test will be used to determine your $\dot{V}O_2max$ and also the workload required to elicit specific percentages of $\dot{V}O_2max$ for use in later Experimental Visits.

If you are not eligible after this screening visit based on your health, you will be informed of this and unfortunately you will not be able to participate further in the study. If you are eligible for inclusion in the study then we will invite you to participate in the Experimental Visits (summarised in the figure below). Between this screening visit and the subsequent Experimental Visits (~1-2 weeks), we will ask you to maintain your normal habitual diet and to wear an accelerometer for 7 days (to allow us to measure your physical activity level). We will then ask you to complete a questionnaire asking about your physical activity level over this 7 day period. This information will be used to allocate you to either a ‘physically active’ or a ‘physically inactive’ group.

During the 48 hours prior to each Experimental Trial and until the end of each respective trial (~3.5 days) we would ask you to do the following:

- Refrain from vigorous exercise and alcohol consumption
- Record your dietary intake (food and drink) so we can analyse your caloric and macronutrient intake
- Wear an accelerometer device on your belt so we can measure your physical activity level
- Wear a continuous glucose monitoring device

Between each Experimental Visit (~1-2 weeks) you must maintain your normal habitual diet and activity habits. Prior to Experimental Trials 2, 3 and 4 we would ask you to repeat as closely as possible the dietary intake and physical activity levels that you completed prior to Experimental Trial 1.

Visits 2 to 5 – Experimental Visits

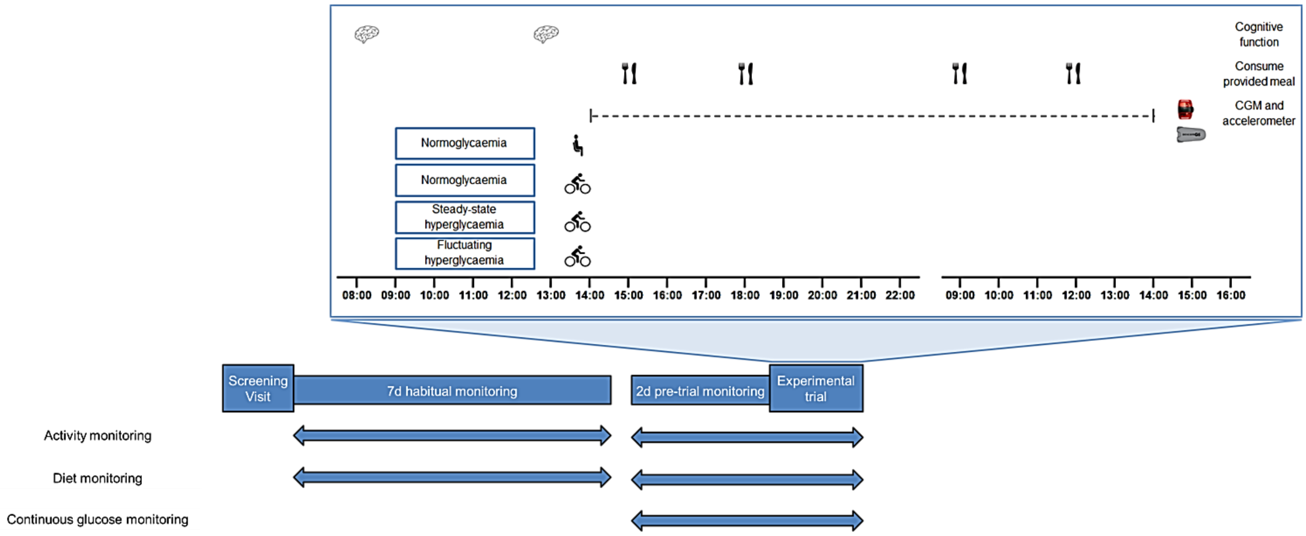

Summary of the experimental design.

For each of these visits we ask you to come to the School of Sport, Exercise and Rehabilitation Sciences at the University of Birmingham between 7 and 8 am, having not eaten or drunk anything except water since 10 pm the previous night. It is very important that you do not eat breakfast or drink anything other than consuming a glass of tap water upon waking.

Upon entering the laboratory, body composition (height, weight, skinfold assessment, waist circumference) will be assessed, and a cannula (small plastic tube) will be placed into a vein in each arm (one for infusion of glucose, one for blood sampling). The following protocol will then commence. Firstly, we will take resting blood samples, followed immediately by assessment of cardiovascular response (heart rate, blood pressure, blood oxygenation), motor function and cognitive function (using an iPad based system). Next, a baseline blood sample (10 ml) would be collected, followed immediately by 1 of 4 interventions (described below) and will continue for 5-hours. Small (3-5ml) blood samples may be drawn up to every 30 minutes during this intervention. After 4 hours 15 minutes of the intervention, a second 10ml blood sample will be collected and you will begin a moderate-intensity exercise bout (45 minutes of cycling at 70% of your HRmax recorded during the screening visit). Upon completion of the exercise bout, a third 10ml blood sample will be collected, the intervention will end, and a 30 min rest period will begin. Urine samples will be collected, and cardiovascular response (heart rate, blood pressure, blood oxygenation), motor function and cognitive function (using an iPad based system) and perceptions of exertion (using a standardised questionnaire) will be assessed at various time points throughout each trial (please see figure). After the rest period, you will be fed a meal, and the catheters will be removed.

Before leaving the laboratory, we will provide you with an evening meal, as well as breakfast and lunch for the following day. You will continue to record your dietary intake, and you will continue to wear the accelerometer and continuous glucose monitor overnight until around 3 pm the following day, after which they can be removed, marking the end of the trial.

You will undergo all 4 Experimental Visits and they will be conducted in a randomised order. These visits will be identical except for the glycaemic intervention that will be used prior to and during the exercise bout, which will proceed as follows:

> **Trial A (normoglycaemia):** This will involve no glycaemic intervention and you will remain in a rested state throughout all subsequent procedures.

Trial B (steady high blood glucose levels): This will involve a square-wave hyperglycaemic clamp with continuous glucose infusion so as to establish a steady glucose profile.

Trial C (fluctuating high blood glucose levels): This will involve repeated glucose infusions so as to cause multiple fluctuations in glucose level.

Trial D (normoglycaemia, no exercise control trial): This will be identical to trial A, except there will be no exercise bout.

What are the possible disadvantages and risks of taking part in this study?

Exercise test. You will experience fatigue towards the end of the exercise tests. This will however be short lived and you should fully recover within a few minutes. However during such vigorous exercise there is a very minimal risk of a cardiovascular event (e.g., a heart attack). The absolute risk that an acute cardiovascular event will occur during vigorous exertion has been estimated to be 1 in 2,600,000 hours of exercise. In addition, intense physical activity may increase the chance of you fainting, or experiencing a stress fracture. However, as you are healthy and accustomed to physical activity the risk is extremely small and these procedures are regularly conducted within the laboratory. At least one investigator trained in first aid procedures (St. Johns Ambulance) and CPR will be 'on hand' during all exercise testing sessions.

***Continuous glucose monitoring*.** This is a small wireless flexible plastic probe that sits just under the skin in your abdomen and allows us to measure blood glucose levels. The probe is inserted using a small spring-loaded needle that very rapidly inserts the flexible plastic probe under the skin. The probe is directly connected to a smooth plastic device which is ~2 cm x 2 cm in dimensions and sits closely against the skin. The probe is worn continuously for 4.5 days, after which it is removed, disconnected from the device, and discarded.

You may experience a slight discomfort as the plastic catheter is inserted under the skin of your abdomen and a small bruise may appear at the site afterwards. However, this risk is very minimal, and the study researchers are fully-trained and highly experienced with such devices.

***Blood Sampling*.** You may experience a slight discomfort as the cannula is inserted for the blood collection and a small bruise may appear at the site afterwards. There is also a small risk of a needle stick injury and infection. However, these risks are minimal as the researchers are well trained and experienced in the safe conduct of this procedure.

Blood volumes. The total amount of blood that we would take is a **maximum of 500 millilitres**. This equates to less than 1 pint. It is safe to lose this amount of blood, but you should not donate blood for the next two months.

Glycaemic intervention. You may experience slight discomfort during this procedure characterised by nausea and headaches due to changing blood glucose levels. However, this discomfort is very minimal and short-lived (<30 minutes), and the researchers are well trained and experienced in the safe conduct of this procedure.

Electrocardiography (ECG). This is a non-invasive, painless and safe procedure with which to record the electrical activity of the heart. This procedure will allow us to record your heart rate throughout each study day. The only slight discomfort that may arise from this procedure is during the removal of the electrodes which would be taped to your chest. Such discomfort is similar to removing a bandage/plaster and would be extremely short-lived. There are no known risks of this non-invasive procedure.

Comfort and follow-up. During each study day you will be asked to lie in a bed or sit in a chair for up to 8 hours maximum, which may be uncomfortable. The laboratory is equipped with fully adjustable beds for that purpose. WC and washing facilities are also available and they can be used without restriction throughout the study. You will also remain fasted during the study day. We will ensure that you are well hydrated throughout, and we will provide a meal as soon as the study day is finished. There will be a follow up by phone and/or email after each study day to ensure you have not experienced any adverse reactions to any of the procedures.

Confidentiality:

All of your records and data will be kept securely (i.e., locked filing cabinet and/or password protected computer files) and confidential with access restricted solely to members of the immediate research team. Personal data would be stored for a maximum of 6-12 months after the completion of the study. All research data will be labelled with a code rather than your name, and only members of the immediate research team will have access to the link between the code and your name.

We would perform some analyses on the samples soon after you visited. We would also store some samples up to 10 years after the study to make further analyses on as we develop new analytical methods in the laboratory, so as to gain additional information. It is possible that a small portion of blood samples collected during your visits may be sent to our colleague, Dr Jacob Haus (Assistant Professor, Department of Kinesiology and Nutrition, University of Illinois at Chicago), for specialised analysis not currently possible within our laboratory. Dr Haus would not receive any personal information about yourself and will therefore be blinded to your identity so as to preserve your anonymity. After 10 years maximum, all research data (including electronic data, questionnaires and any remaining samples) will be destroyed. The result of this study is expected to be published in a scientific journal, but your name will never be published.

What will happen if the screening results show anything abnormal?

In this case we would contact and inform you immediately. Since our study is designed for healthy individuals, you would not be able to participate in this study.

What will happen to the results of the research study?

The result of this study is expected to be published in a scientific journal, and we can send you a copy if you are interested.

What are my rights?

It is your choice whether or not you wish to take part in this study. If you wish to take part in this study, you will be asked to sign a consent form. You are reminded that if you decide to take part in the study, you are still free to withdraw from the study at any time without provision of reason. That said, your deadline for withdrawal of your data from the study will be on the morning of the final testing day. If you complete all trials, you will then not be eligible to withdraw your data. Should you choose to withdraw, please let us know via email at your earliest opportunity and we will contact you to confirm removal of your data from the study. Data will not be retained from participants who withdraw from the study.

Requests for a copy of the results attained will be honoured but only after the study is completed and published in a peer-reviewed scientific journal.

What happens if something goes wrong on the day of the trials?

All procedures have been included within the University of Birmingham Liability Insurances and if you are harmed in any way by taking part in this research project your normal rights apply and you may have grounds for legal action.

If you have any complaints regarding the way you have been treated or anything else relating to the study, you can contact Dr Sean Jennings who is independent from the research team and will investigate the matter fully:

Dr Sean Jennings
Research Governance and Ethics Manager, University of Birmingham, Birmingham B15 2TT
Phone: +44 (0) 121 415 8011
Email: researchgovernance@contacts.bham.ac.uk

What can I do if I would like more information?

If you have any further questions about the study, please feel free to contact any of the study investigators directly:

Mr Steven Carter
Doctoral Researcher, and Chief Investigator
School of Sport, Exercise, & Rehabilitation Sciences
University of Birmingham, Edgbaston, Birmingham B15 2TT
Phone: +44 (0) 7858270668
Email: sxc752@bham.ac.uk

Dr Thomas Solomon
Senior Lecturer & EU Marie Curie Research Fellow, and Principle Investigator
School of Sport, Exercise, & Rehabilitation Sciences
University of Birmingham, Edgbaston, Birmingham B15 2TT
Phone: +44 (0) 121 415 8609
Email: t.solomon@bham.ac.uk

If you would like to discuss the study with an independent source of advice before deciding whether or not to participate, please feel free to contact Dr Sean Jennings who is unconnected to the study and will help answer any questions you may have:

Dr Sean Jennings
Research Governance and Ethics Manager, University of Birmingham, Birmingham B15 2TT
Phone: +44 (0) 121 415 8011
Email: researchgovernance@contacts.bham.ac.uk

What should I do if I would like to participate in the study?

Thank you very much for your time reading this information sheet and your interest in our study. If you would like to participate in the study, please contact Mr Steven Carter who will invite you to a screening visit. During this visit we will provide further explanation of the project, as well as answer any questions you may have. At this point, if you are happy to participate in the study, you will be asked to complete an informed consent form to confirm that you are happy to participate in this study. You will be asked to keep a copy of this information sheet and the signed consent forms.

Mr Steven Carter
Doctoral Researcher, and Chief Investigator
School of Sport, Exercise, & Rehabilitation Sciences
University of Birmingham, Edgbaston, Birmingham B15 2TT
Phone: +44 (0) 7858270668
Email: sxc752@bham.ac.uk
