# Supplementary material for: Plasma FGF21 concentrations are regulated by glucose independently of insulin and GLP-1 in lean, healthy humans
Source: PeerJ. 2022 Jan 19;10:e12755. doi: 10.7717/peerj.12755 (PMC8783558; doi:10.7717/peerj.12755)
Supplement: Supplemental Information 3 [file peerj-10-12755-s003.pdf]

RESEARCH PROTOCOL

Title: Chronic Hyperglycemia, Advanced Glycation Endproducts and Peripheral Insulin Resistance with Type 2 Diabetes

Principal Investigator:

Jacob M. Haus, PhD
Assistant Professor
Department of Kinesiology and Nutrition
University of Illinois at Chicago
1919 W. Taylor St (MC 517)
Chicago, IL 60612

Ph: 312-413-1913
Fx: 312-413-0319
email: hausj@uic.edu

Co-Investigators: Terry Unterman, MD; University of Illinois at Chicago

Date: 4.30.17_v5

ABSTRACT

Chronic hyperglycemia results in protein glycation and metabolic stress which can generate free radicals and further damage glycated tissues. This process is called advanced glycosylation and results in the formation of advanced glycation endproducts (AGEs). AGE deposits have been found in atherosclerotic plaques, vascular smooth muscle, skeletal muscle, adipose and in myocardial tissues. AGEs play a critical role in the pathogenesis of chronic diabetic complications by altering cellular structure and function. However, the critical barrier to progress in this area is that the precise mechanisms of AGE induced tissue damage are poorly understood in humans and organ specific mechanistic investigation is warranted. Recently, the receptors for advanced glycation endproducts (RAGE) were found to disrupt glucose metabolism in rodent skeletal muscles and adipose tisue suggesting that these compounds play a role in the pathogenesis of type 2 diabetes. The proposed work will improve the mechanistic understanding of AGE/RAGE mediated molecular events and how they contribute to tissue specific pathophysiology such as inflammation and peripheral insulin resistance. *The primary goal* of this research is to explore the relationship between glycation endproducts in skeletal muscle, adipose tissue and glucose metabolism. We will test our hypotheses by performing hyperinsulinemic-euglycemic clamps and muscle and adipose biopsies in type 2 diabetic subjects and controls. In addition, we will study the proteins in the muscle and adipose tissues to determine how the advanced glycation has damaged these proteins. We expect that greater amounts of glycation endproducts found in skeletal muscles and adipose tissue are related to disruptions in glucose metabolism and that proteins important for glucose metabolism have been damaged by the glycation process. The proposed work is innovative as this is the first human investigation to study AGE/RAGE mediated cellular events in skeletal muscle and adipose tissue as they pertain to the development of insulin resistance and long-term diabetic complications. This work will elucidate a novel mechanism of AGE/RAGE-induced insulin resistance that manifests with chronic hyperglycemia, inflammation and oxidative stress. New roles for AGEs in disease pathology are being revealed as glycosylation of mitochondria may inhibit cellular respiration and glycated contractile elements correlate with reduced muscle function and triglyceride storage. These data will provide the platform for future investigations to isolate and quantify specific intracellular proteins such as mitochondrial membranes and metabolic enzymes in the ongoing search for the mechanisms of hyperglycemia induced insulin resistance, oxidative stress and tissue damage. This study capitalizes on interdisciplinary translational science via our ability to couple *in vivo* measurements of peripheral insulin resistance with skeletal muscle and adiposetissue sampling, quantification of AGEs and molecular exploration of insulin resistance signaling events. Furthermore, the Applicant and the assembled investigative team is well prepared to undertake the proposed research, because in addition to expertise with the clinical and basic science procedures, this work will be carried out in a research environment that is conducive to its successful completion. The controlled research environment of the Clinical Research Center (CRC) is central to the completion of the proposed clinical and metabolic procedures while the UIC CCTS Core services will provide the equipment and technical personnel for the completion of the proposed analytical measures.

SPECIFIC AIMS

Protein glycation alters cellular structure and function in patients with diabetes mellitus. These modifications occur in the form of advanced glycation endproducts (AGEs) which arise from chronic hyperglycemia, dyslipidemia, and oxidative stress. AGEs form via the Maillard Reaction when reducing sugars react spontaneously with free amino groups of proteins (1). Accretion of AGEs reflects cumulative metabolic stress rather than short-term glycemic control and are strong predictors of future diabetic complications (2). Recently, AGEs in skeletal muscle of mice were found to inhibit insulin action through a novel signaling pathway that induces the receptor for AGE (RAGE) (3). Further, RAGE has been found in adipose tissue and was mechanistically linked to adipose inflammation. These data suggest AGEs play a unique role in the development and progression of skeletal muscle and adipose insulin resistance through AGE/RAGE molecular interactions. However, the role of AGE/RAGEs in skeletal muscle and adipose of human subjects with type 2 diabetes has not been investigated. *The primary objective* of this application is to explore the relationship between AGEs in human skeletal muscle and adipose tissue and peripheral insulin resistance. *The central hypothesis* is that AGEs found in skeletal muscle are associated with the degree of peripheral insulin resistance and that markers of AGE/RAGE signaling will correlate with impaired insulin signaling. The *rationale* for the proposed research is that once it is known how AGEs in skeletal muscle and adipose contribute to peripheral insulin resistance, therapeutic strategies such as lifestyle intervention and/or pharmacology that target skeletal muscle and adipose AGE/RAGE signaling can be implemented. Given new data linking AGE/RAGE to impaired insulin action, this study is both timely and clinically significant. Our proposed studies will expand the knowledge of skeletal muscle and adipose biology into a novel area of study that focuses on the mechanisms of AGE/RAGE induced peripheral insulin resistance and will have important implications to other i tissues such as vascular smooth muscle, endothelium, heart,,and liver. We plan to test the following specific aims:

Specific Aim 1: Evaluate the relationship between skeletal muscle and adipose AGEs and RAGE and peripheral insulin resistance. The *working hypothesis* for this aim is that AGE/RAGE is elevated in the skeletal muscle and adipose of individuals with type 2 diabetes compared to age matched and young controls, and that the degree of insulin resistance correlates with AGE/RAGE. The *approach* used to test this hypothesis will be to conduct euglycemic-hyperinsulinemic clamps to access peripheral insulin action and obtain skeletal muscle and adipose samples from 40 healthy control subjects and 40 obese type 2 diabetic subjects. AGEs will be quantified via tandem mass spectrometry coupled with high performance liquid chromatography (LC-MS/MS) and RAGE quantified by immunodetection methods We expect that AGE/RAGE will be greater in diabetic subjects and that insulin resistance will positively correlate with AGE/RAGE appearance in skeletal muscle tissue.

Specific Aim 2: Determine the effects of type 2 diabetes on AGE/RAGE and insulin signaling events in skeletal muscle. The *working hypothesis* for this aim is that activation of AGE/RAGE signaling is greater in skeletal muscle of diabetic subjects compared to controls, and that greater expression of these signaling molecules will inversely correlate with signaling related to insulin action. The *approach* used to test this hypothesis will be to identify differences in AGE/RAGE and insulin signaling protein expression and activation in tissue obtained from control and diabetic subjects at baseline and during insulin-stimulated conditions. We will probe for differences in protein expression of RAGE, insulin receptor substrate-1 (IRS-1), protein kinase-B (AKT), protein kinase C-alpha (PKCα) and nuclear factor kappa-B (NFκB) via standard molecular biology techniques. We expect RAGE expression will be upregulated, while proteins in the insulin signaling pathway will be down-regulated in diabetics compared to normal controls.

SIGNIFICANCE AND INNOVATION

Cardiovascular disease (CVD) is the major cause of morbidity and mortality associated with diabetes. Patients with diabetes have a mortality rate from CVD two times greater than the general population (4-6). There is increasing evidence that both AGEs and insulin resistance, which both precedes and predicts the development of type 2 diabetes and risk of CVD, play a pivotal role in the progression of diabetic atherosclerosis. Serum AGE levels are elevated in diabetic patients with coronary heart disease (CHD) and correlate strongly with CHD severity. AGE deposits have been found in atherosclerotic plaques, vascular smooth muscle and skeletal muscle,adipose, and in myocardial tissues (7; 8). AGEs play a critical role in the pathogenesis of chronic diabetic complications by altering cellular structure and function. However, the critical barrier to progress in this area is

that the precise mechanisms of AGE induced tissue damage are poorly understood and organ specific mechanistic investigation is warranted. This work will improve the mechanistic understanding of AGE/RAGE mediated molecular events and how they contribute to tissue specific pathophysiology such as inflammation and peripheral insulin resistance. Once the mechanisms of AGE/RAGE-induced skeletal muscle and adipose tissue damage are elucidated, strategies for the treatment and prevention of AGE induced tissue damage can be advanced in additional organ systems thus leading to the improved therapeutic management of diabetes and long-term complications.

The proposed work is innovative as this is the first human investigation to study AGE/RAGE mediated cellular events in skeletal muscle and adipose tissue as they pertain to the development of insulin resistance and long-term diabetic complications. This work will elucidate a novel mechanism of AGE/RAGE-induced insulin resistance that manifests with chronic hyperglycemia and oxidative stress. The investigation of glycated proteins in skeletal muscle and adipose tissue is in its infancy. This area holds vast potential as skeletal muscle and adipose are primary glucose disposal tissues and a primary site of oxidative stress. New roles for AGEs in disease pathology are being revealed as glycosylation of mitochondria may inhibit cellular respiration and glycated contractile elements correlate with reduced muscle and adipose function. These data will provide the platform for future investigations to isolate and quantify specific intracellular proteins such as mitochondrial membranes and metabolic enzymes in the ongoing search for the mechanisms of hyperglycemia induced insulin resistance, oxidative stress and tissue damage. This study capitalizes on our ability to couple in vivo measurements of peripheral insulin resistance with skeletal muscle and adipose tissue sampling, quantification of AGEs and molecular exploration of insulin resistance signaling events. Use of the euglycemic-hyperinsulinemic clamp for the quantification of insulin resistance is the gold-standard technique. The quantification of AGEs via established mass signatures determined by tandem mass-spectrometry has significant advantage over other methodologies because the sensitivity of the assay allows for small tissue amounts of ≤ 10mg. Use of stable isotope amino acid standards are also the gold standard for quantification and will enhance sensitivity and accuracy of the AGE measures.

AGEs and Skeletal Muscle: AGEs have been studied in collagenous tissues, urine and serum; however, sparse data are available in skeletal muscle and adipose tissue. This is surprising given the role that skeletal muscle and fat plays in glucose disposal and its function as a primary insulin-sensitive tissue. At the present time, we know of only three studies examining AGEs in human skeletal muscle (8; 15; 16) and one is adipose. Post-mortem analysis of human skeletal muscle tissue from one young (15 y) and one old (82 y) individual (15) showed the AGE, pentosidine, could not be detected in the young individual but was measurable in the old at relatively substantial levels (~ 40 mmol pentosidine/ mol collagen) in comparison to other tissues. Ruster *et al*. (16) studied 81 healthy controls and 41 individuals with fibromyalgia for the AGE, carboxymethyllysine (CML), from muscle histology preparations and found that CML was present in all subjects and staining intensity was less in healthy controls versus fibromyalgia. In addition, previous work performed by the Applicant in healthy aging men and women shows that AGEs are significantly elevated in older sedentary individuals compared to young (8). Further, Alt and colleagues (17) examined AGE formation in the skeletal muscle of diabetic and control rats. As expected, diabetic rats had a three-fold increase in muscle AGE concentration. Similar findings

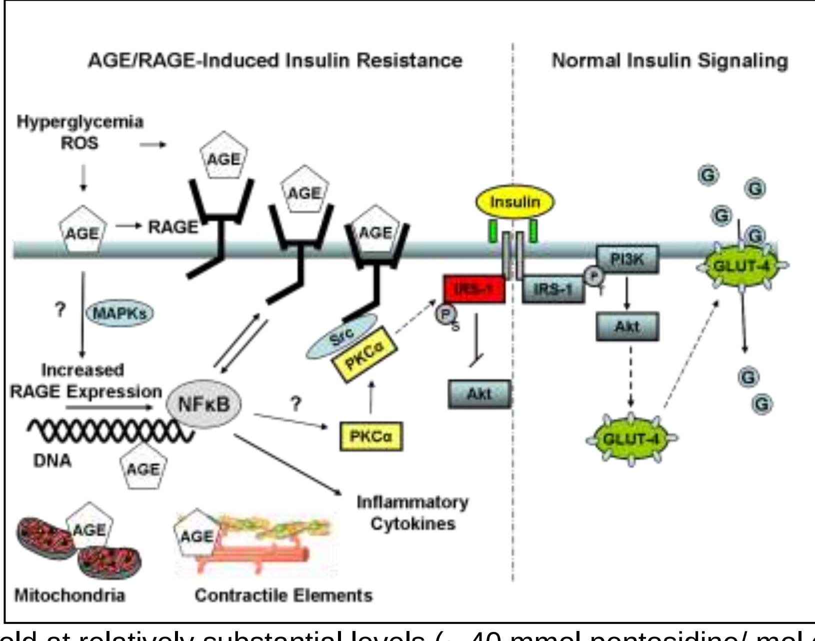

Fig.1. Schematic representation of AGE/RAGE-induced insulin resistance in skeletal muscle. Under normal conditions (right panel), the insulin receptor phosphorylates insulin receptor substrate-1 (IRS-1) proteins at tyrosine residues site which allow IRS-1 to bind to phosphatidylinositol 3-kinase (PI3K) and subsequently Akt/protein kinase B. AKT then initiates the translocation of GLUT-4 vesicles to the plasma membrane allowing glucose uptake to occur. Conversely, when uncontrolled diabetes results in chronic hyperglycemia and formation of reactive oxygen species (ROS); advanced glycation endproducts (AGEs) form on tissue and plasma proteins. AGEs also form intracellularly on DNA, mitochondria and contractile proteins from increased glucose flux. AGE formation results in expression of the receptor of advanced glycation endproducts (RAGE) via cellular stress pathways that may include the mitogen activated protein kinases (MAPKs) and other inflammation pathways mediated by nuclear factor kappa-B (NFκB). Upregulation of RAGE via NFκB provides increased cell surface expression of RAGE and increases the number of binding sites for circulating AGE ligands. AGE/RAGE ligation initiates the association of RAGE, Src kinase and protein kinase C-alpha (PKCα) which then inhibits insulin signaling by phosphorylating IRS-1 at specific serine residues such as Ser307, 270, and 612. Phosphorylation of Ser307 has been shown to be a negative regulator of insulin signaling resulting in decreased insulin stimulated glucose uptake.

were observed by Brown and others (18) in rat skeletal muscle; whereas Thornalley *et al.* (19) were able to detect a total of 9 different AGEs in both control and diabetic rat skeletal muscle with LC-MS/MS. Recently, AGEs in the skeletal muscle of mice have been shown to inhibit insulin action through a novel multimolecular complex that induces the receptor for AGE (RAGE) (3). These data suggest that AGEs may play a role in the development of skeletal muscle insulin resistance through its cell surface receptor RAGE (Fig.1.) However, the role of AGEs in subjects with pre-diabetes (impaired glucose tolerance, IGT) or type 2 diabetes has not been investigated despite evidence that weight gain is associated with increased AGEs, RAGEs and oxidative stress in older obese individuals (20).

PRELIMINARY DATA.

The Applicant has extensive research experience dealing with the study of human subjects with a strong emphasis on clinical investigations dealing with insulin resistance and skeletal muscle function. Descriptions of these studies and data are presented to provide a foundation for the proposed project and outline the Applicants experience with the proposed measures and ability to successfully conduct human clinical research.

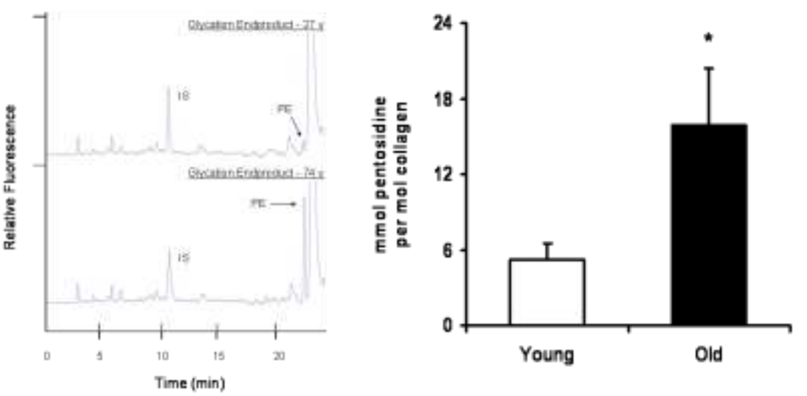

Fig. 2. Representative HPLC chromatograph for the quantification of the AGE, pentosidine, in human skeletal muscle. Pentosidine was quantified in skeletal muscle tissue collected from both young and old individuals. Older individuals displayed greater tissue concentrations of pentosidine, * $p < 0.05$.

1.) Detection of AGEs in Human Skeletal Muscle. In previous work, the Applicant examined the hypothesis that AGEs contribute to the loss of muscle strength, decline in physical performance and disability in older adults. These data show that the AGE, pentosidine, is significantly elevated in the skeletal muscle of older adults (70±1 yrs) with concomitant sarcopenia (8). These data provide support that AGEs are present in human skeletal muscle and may contribute to muscle dysfunction. These data are important to this proposal because, the risk of diabetes development is greatest in older sedentary adults. As such, the ~ 3.5 fold increase in pentosidine seen with this population provides evidence that AGEs may play a significant role in AGE-induced skeletal muscle insulin resistance.

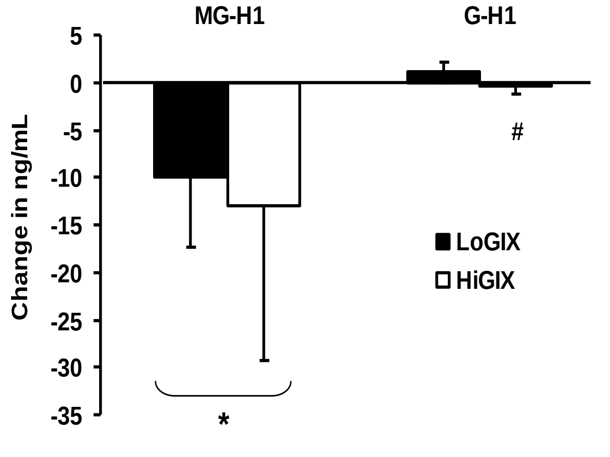

Figure 3. Change in methylglyoxal and glyoxal-derived hydroimidazolone-AGEs following an exercise training and glycemic index diet lifestyle intervention. LoGIX = low glycemic index plus exercise, HiGIX = high glycemic index plus exercise. * indicates a significant effect of time and # indicates a significant effect of group.

2.) Methylglyoxal and glyoxal derived hydroimidazolones-AGEs are reduced with exercise training and a low-glycemic index diet. Recent funded work performed by the applicant demonstrates an intensive lifestyle modification reduces AGEs in obese adults with impaired glucose tolerance (IGT). Aldehydes derived from glucose, such as methylglyoxal (MG-H1) and glyoxal-derived hydroimidazolone (G-H1), react with proteins resulting in glycation that can yield AGEs. Twenty-seven obese subjects with IGT, (mean ± SEM; age: 65 ± 1 y; BMI: 35.3 ± 0.8 kg/m^2), underwent a 12-wk aerobic exercise-training intervention (1 h/d, 5 d/wk, 85% of HRmax) while randomly assigned to receive either a low-GI diet (LoGIX; n = 13, 40 ± 0.3 Units) or a high-GI diet (HiGIX; n = 14, 80 ± 0.6 Units). Free plasma MG-H1 and G-H1 were measured by LC-MS/MS. MG-H1 was reduced in both groups, however the HiGIX group demonstrated greater reductions in G-H1 compared to LoGIX. These data suggest that regular aerobic exercise and a controlled diet can reverse the formation of AGEs which may lead to reduced risk of future diabetes and diabetic complications. Future studies will examine mechanisms of these changes.

3.) Intensive insulin therapy attenuates systemic Nepsilon-carboxymethyllysine and reduces HbA1c in poorly controlled African American patients with type 2 diabetes

The applicant previously evaluated the change in fasting plasma glucose (FPG), hemoglobin A1c (HbA1c), and plasma Nepsilon-carboxymethyllysine (CML) concentrations (a common AGE) in poorly controlled African American type 2 diabetic patients (n=6) before and after a 3-month intensive insulin therapy regimen. The treatment goal was near normalization of mean plasma HbA1c levels of 6.5% or less. The intensive insulin regimen consisted of multiple daily injections of lantus/apidra with frequent titration. Mean subject age (±SEM) was 54±3 yrs, body mass index 42±3 kg/m2, while baseline FPG and HbA1c were 259±32 mg/dl and 11.0±0.7%, respectively. Following the 3 month intervention period plasma CML fell from 11.0±1.0 to 9.8±0.5 ng/ml (P=0.03). These data provide evidence supporting the notion that therapeutic regimens capable of reducing HbA1c levels in poorly controlled type 2 diabetic patients can significantly attenuate the production of AGEs and reduce the risk of cardiovascular disease.

EXPERIMENTAL DESIGN AND METHODOLOGY

Overview. This research will involve the prospective study of 40 lean (BMI: 18 - 25 kg/m^2) healthy and 40 obese (BMI: 26 - 40 kg/m^2) diabetic men and women (n = 20 men and 20 women in each group) ages 18 – 70 yrs old. Insulin sensitivity will be determined by hyperinsulinemic euglycemic clamp and glycation endproducts will be measured from skeletal muscle and adipose biopsy. Additional metabolic tests such as indirect calorimetry, oral glucose tolerance test (OGTT) and dual energy x-ray absorptiometry (DEXA) will be performed to characterize the subject's metabolic health and demographics. Lean healthy control subjects will be both age and gender matched to subjects in the type 2 diabetes group. AGEs will be quantified via LC-MS/MS and signaling pathways will be examined by standard molecular biology techniques. Table 1. describes the subject commitment by indicating the number of visits, as well as the composition and time of the visits. A study flow diagram is presented in Figure 4. and a time-line diagram of the hyperinsulinemic-euglycemic clamp/muscle biopsy procedure is presented in Figure 5.

Figure 4. Study Flow Diagram

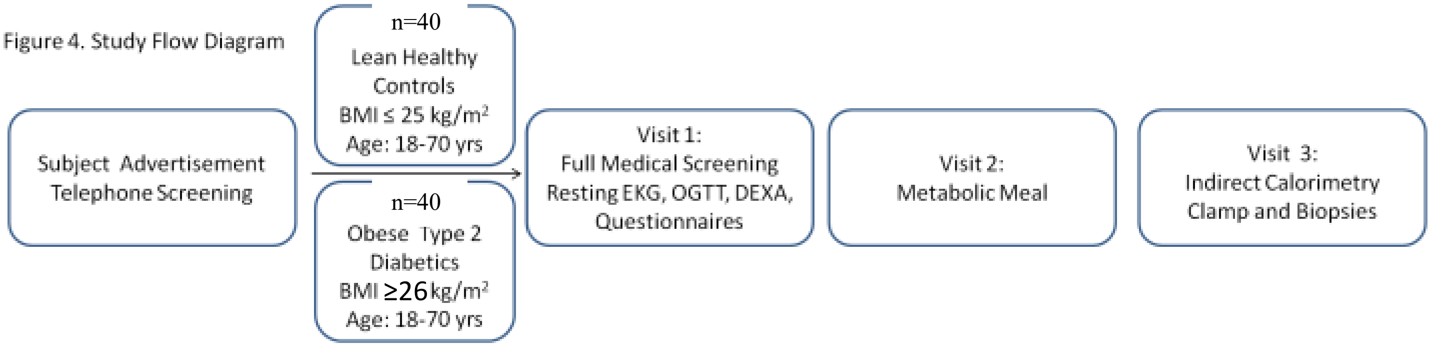

Subject Screening: Potential subjects will learn of the study through community posted advertisements and will respond by telephone if interested. During this initial phone call, the study will be described in detail to potential participants and an initial pre-screening will take place. During this time, we will obtain information about the potential subject's name, contact information, age/date of birth, body mass index (BMI), smoking and physical activity status, current medical conditions, allergies, health status and medications. This information is used for initial screening purposes only. In the event the subject should not meet initial screening criteria, the information collected will be destroyed. Those subjects who meet the initial screening criteria and express willingness to participate by providing verbal consent will be scheduled for a complete medical screening in the CRC. Approximately one week prior to the screening visit, potential subjects will be mailed an information packet that will include a copy of the informed consent for their review and detailed instructions on how to travel to UIC, where to park and guidelines about what constitutes- and how to eat a well balanced meal that contains at least 150 to 200 grams (g) of carbohydrate per day for 3 days before the screening visit. In addition, subjects will be asked not to consume caffeine or exercise strenuously for at least 12 hours before the screening visit. Subjects will be asked to report to the CRC after an overnight fast of 10-12 hrs. Upon arrival the subject will have the study explained to them and will be permitted to ask questions regarding the study procedures. Written informed consent will be obtained during this initial visit. Subjects are screened based on the inclusion/exclusion criteria. A questionnaire is completed by the subject regarding past and current medical conditions. The study physician (Terry Unterman, MD), or nurse practitioner then reviews the information provided and performs a full medical

history and physical exam, including a 12-lead resting electrocardiogram. The resting ECG will be reviewed by the study physician prior to subject enrollment. A 75-gram Oral Glucose Tolerance Test (OGTT, duration 180 min, see details below) is also performed to screen for abnormal glucose tolerance in healthy control subjects and confirm type 2 diabetes in diabetic subjects. During the OGTT, the subject will be asked to complete questionnaires pertaining to their dietary and activity habits. Fasting baseline blood samples will be obtained to determine lipid profiles, liver and hematological function. In addition, female subjects that are pre-menopausal will be asked to provide a spot urine sample for a pregnancy test. Following the OGTT, the subjects will be provided with a snack and will then undergo a whole body DEXA scan (see below) for measurement of body composition in the Integrated Physiology Lab located on the first floor of the Disability, Health and Social Policy (DHSP) building, located at 1640 West Roosevelt Road. This test can also be performed on visit 2 or 3 depending upon the subject's schedule. After which, the subjects will be discharged. If any blood work or test results are clinically significant, the patient will be referred to their own personal physician for additional evaluation prior to further consideration for participation in this study. Each subject will be reviewed by the PI and Co-Investigator and study physician, Dr Terry Unterman, M.D. who will assist with the recruitment efforts of diabetic subjects at UIC Medical Center. The PI will determine final subject eligibility for study participation and reserves the right to modify the order of testing events to facilitate subject availability and study integrity.

ResearchMatch.org will be also be used as one of the recruitment tools for this research study. ResearchMatch Volunteers will be contacted through ResearchMatch.org. Included with this submission is a study recruitment message that will be sent to potential study volunteers. Once the ResearchMatch volunteers agree to be contacted and his/her contact information is released to through ResearchMatch, the potential subjects we will follow the same procedures as discussed above, starting with the initial prescreening.

Each subject will be reviewed by the PI and study physician, Dr Terry Unterman, M.D. who will assist with the recruitment efforts of diabetic subjects at UIC Medical Center. The PI will determine final subject eligibility for study participation.

Inclusion Criteria

- The study is open to males and females aged 18 – 70 years.
- Potential subjects in both groups will be fluent (written and verbal) in English.
- Potential subjects to the Lean Healthy Control Group will have BMI: 18 - 25 kg/m^2 and be healthy.
- Potential subjects to the Type 2 Diabetes Group will be newly diagnosed with Type 2 diabetes, have a BMI: 26 - 40 kg/m^2 and will be currently treated with only lifestyle intervention to eliminate potential effects of medication (i.e. insulin, biguanides, thiazolidinediones, sulfonylureas or other secretagogues, alpha-glucosidase inhibitors or peptide analogs) and be free of other existing disease that may influence the primary outcome variables.

Exclusion Criteria

<u>LEAN HEALTHY CONTROL GROUP</u>

- abnormal glucose tolerance (American Diabetes Association and WHO criteria)
- a BMI of < 18 and > 25 kg/m^2
- smokers/smokeless tobacco use
- evidence of overt type 1 and 2 diabetes
- evidence of cardiovascular, cerebrovascular, liver, renal, hematological, thyroid disease, cancer or other metabolic disease by self report or at screening visit
- hypertriglyceridemic (>400 mg/dl) or hypercholesterolemic
- subjects must be weight stable (<2 kg weight change in the past 6 months)

- free of any additional contraindications for participation
- all premenopausal women will receive a point-of-care pregnancy test at screening or prior to the DEXA scan (if the DEXA occurs at a time different than the screening visit) and will be excluded from participation if pregnant. All other women must be postmenopausal for at least 1 year, and must agree to remain off hormone replacement therapy for the duration of the study.

OBESE TYPE 2 DIABETES GROUP

- a BMI of < 26 and > 40 kg/m^2
- smokers/smokeless tobacco use
- evidence of exogenous insulin therapy as treatment for diabetes
- evidence of cardiovascular, cerebrovascular, liver, renal, hematological, thyroid disease or cancer by self report or by screening visit
- hypertriglyceridemic (>400 mg/dl) or hypercholesterolemic
- subjects must be weight stable (<2 kg weight change in the past 6 months)
- free of any additional contraindications for participation
- all premenopausal women will receive a point-of-care pregnancy test at screening or prior to the DEXA scan (if the DEXA occurs at a time different than the screening visit) and will be excluded from participation if pregnant. All other women must be postmenopausal for at least 1 year, and must agree to remain off hormone replacement therapy for the duration of the study.

Metabolic Control Period: For 3 days prior to the metabolic testing day, subjects will be counseled to eat a balanced diet that contains at least 200 grams (g) of carbohydrate per day. During these days, subjects will be asked to record the time, content and volume of foods and beverages consumed using dietary recall questionnaires. Assessments of peripheral insulin sensitivity and skeletal muscle/adipose biopsy will be performed in the CRC following the 3 day diet stabilization period. The evening prior to the Clamp procedure, participants will be asked to come to the Applied Health Science Building (AHSB) at ~ 6pm and will be provided a metabolic meal (55% carbohydrate (≥ 250 grams), 35% fat, 10% protein) in the Metabolic Kitchen of the Kinesiology and Nutrition Department. The purpose of this meal is to stabilize muscle and liver glycogen stores; given these are most important factors for determining insulin sensitivity. After meal consumption, the subjects will be required to fast overnight for a period of 10-12 hrs. Subjects will be permitted to drink water only after the metabolic meal. Subjects will be asked to record the time, content and volume of any beverages consumed using a dietary recall questionnaire. During this visit, subjects will be provided supplies and instructions for collection of morning urine (see below). In addition, subjects will be asked to refrain from physical activity outside of their normal activities of daily living for one day prior to metabolic testing. Subjects will be asked to document their physical activity during this period using a physical activity recall questionnaire. Subjects will also be asked to refrain from consuming foods and beverages that contain caffeine for a period of 12 hrs prior to metabolic testing. This approach has been successfully used by the PI and others in studies of insulin sensitivity and metabolism to control for the influence of diet and physical activity.

Metabolic Testing Day:

Initial procedures and Indirect Calorimetry: On the morning of the metabolic tests, the subjects will be asked to collect and document the time their first morning void after 4:00am using a 24-urine container provided to them the evening prior. Subjects will then be asked to arrive at the CRC at ~ 5:30 am at which time they will be asked to rest quietly supine in a darkened room for 30-40 min to return the body to a resting state. After the resting period is completed, baseline substrate metabolism will be measured via indirect calorimetry using the dilated hood technique. Indirect calorimetry will be repeated during the last 30 min of the insulin period of the clamp (see below). The principal of this test is that the molar ratio of oxygen consumed to carbon dioxide produced is used to derive a measure of the relative amounts of substrate that are being oxidized [respiratory quotient (RQ)]. Resting energy expenditure (REE) will be calculated using the Weir equation [54]. Calorimetry

will be corrected for protein oxidation by measurement of urinary urea nitrogen obtained from urine collected from the subject. Total volume and time of collection are recorded and analyzed for urea nitrogen.

Insulin Sensitivity Test: Whole body insulin sensitivity is assessed using the gold-standard hyperinsulinemic-euglycemic clamp procedure (40 mU•m^{-2}•min^{-1} insulin, and 5.0 mM glucose) as described previously and used routinely by the PI (21; 22). The hyperinsulinemic-euglycemic clamp technique was developed in 1966 and has been widely used in diabetes, obesity and metabolic research since. From a mechanistic point of view, this techniques applies a negative feedback principal to the system regulating blood glucose concentration where exogenous insulin is infused to create a hyperinsulinemic plateau of plasma insulin concentrations, while the plasma glucose concentration is kept constant ("clamped") at the euglycemic level (90 mg/dl or 5.0 mM) by means of a variable exogenous glucose infusion. Thus, the rate of glucose infusion required to maintain constant glycemia during the period of constant hyperinsulinemia provides a measure for the net effect of insulin on whole body glucose metabolism. Often, a stable isotope (non-radioactive) dilution technique of a glucose tracer (i.e. [6,6-D2]-glucose, D-glucose labeled with deuterium (heavy hydrogen)) is performed simultaneously with the clamp to measure endogenous glucose output and the rate of glucose disappearance from the blood. In addition, indirect calorimetry (above) and muscle biopsy (below) are also performed with a clamp procedure. In order to perform a hyperinsulinemic-euglycemic clamp test coupled with stable isotope tracers, several technical requirements must be met. Two intravenous lines must be kept patent for the duration of the clamp test, one for the simultaneous infusion of glucose, insulin and glucose-tracer, and the other for frequent blood sampling of arterialized venous blood. In order to avoid potential interference of ipsilateral infusion and blood sampling, contralateral placement of venous catheters is recommended. In addition, 3 precision calibrated infusion pumps, one for glucose, insulin and glucose-tracer respectively, for fine adjustment of infusion rates (0.02-0.15 ml/min) are required. Finally, the glucose concentration in blood samples must be determined rapidly online to facilitate and accurate adjustment of glucose infusion rate.

Procedures for the Hyperinsulinemic-euglycemic clamp: Following a 10-12 h overnight fast a polyethylene catheter is inserted into an antecubital vein for infusion of insulin, glucose, and [6,6-D2]-glucose (non-radioactive D-glucose labeled with deuterium (heavy hydrogen)). A second catheter is inserted retrograde into a dorsal hand vein, and the hand is warmed in a heated box for sampling of arterialized venous blood (23). Once catheters have been placed, basal endogenous glucose production (EGP) is measured using a bolus (3.276 mg•kg^{-1}) infusion of [6,6-D2]-glucose followed by a constant infusion at 0.0364 mg•kg^{-1}•min^{-1} for 120 min (see Supplementary Materials for details) . All infusions are administered via Harvard PhD 2000 precision infusion pumps (Harvard Apparatus, Holliston, MA) which provide sufficient fine gears for minute adjustments to infusion rates. The PI is responsible for maintaining all pumps and infusion rates during the procedure. Blood for pre-clamp glucose, insulin, and glucose tracer kinetics is obtained every 10 min during the last 30-min of the baseline period and the last 30-min of the clamp (See Figure 5). After the baseline period and muscle biopsy (see below) are completed, a primed-continuous infusion (40 mU•m^{-2}•min^{-1}) of human insulin (Humulin-R, Eli Lilly & Co., Indianapolis, IN; see Appendix YY for details) is initiated and maintained for a period of 120 min (referred to as the "clamp" or "insulin" period). Glucose levels are clamped at 5.0 mM (90 mg/dL) by use of a variable glucose infusion (20% dextrose enriched with [6,6-^{2}H]-glucose, See Supplementary Materials for details). Blood glucose is measured every 5 min via YSI glucose-lactate analyzer which delivers rapid results in less than 60 seconds. Blood glucose values are then used in a custom algorithm by the PI to determine changes in glucose infusion to maintain euglycemia. The clamp procedure is completed after the 120 min period of hyperinsulinemia and the insulin muscle biopsy has been obtained (see below). Upon completion, insulin infusion is discontinued while glucose infusion is continued. The necessary duration and the amount of glucose infused post-clamp largely depend upon the insulin sensitivity of the individual. For each subject, plasma glucose will be stable without exogenous glucose infusion before the venous catheters are removed and the subject is released. In addition, the subject will be provided with a post-clamp meal or snack that consists of carbohydrate and protein to further stabilize blood glucose (i.e. juice, sandwich, etc).

Figure 5. Hyperinsulinemic-Euglycemic Clamp Procedure

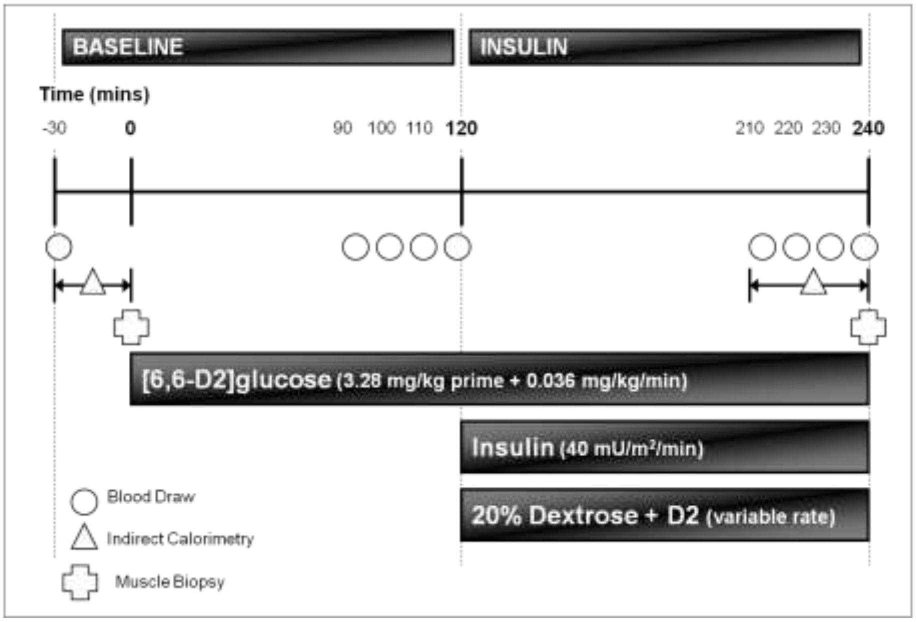

Skeletal Muscle Biopsy: Muscle biopsies will be performed in the Clinical Research Center. Two skeletal muscle and adipose needle biopsies for the quantification of AGEs and signaling proteins will be obtained from each subject. Tissue will be obtained under aseptic conditions from the vastus lateralis muscle and superficial subcutaneous adipose tissue following local anesthetic (Lidocaine HCl 1%) using a 5 mm Bergstrom needle (8; 25-29) during the baseline (Time = -120 - 0 min) and insulin (Time = 120 min) periods of the hyperinsulinemic-euglycemic clamp procedure described above. A small incision is made using a # 11 scalpel to nick the muscle fascia and the Bergstrom muscle biopsy needle is quickly inserted into the muscle belly. Suction is applied using a 140 cc syringe and a small sample of muscle tissue (~ 200 mg) is obtained. Using the same incision, a new Bergstrom biopsy needle is then used to extract ~100 mg of adipose tissue immediately superficial to the vastus lateralis muscle. This approach is commonly used to obtain both tissue types in an effort to reduce subject burden and reduce risk incurred from a separate incision. Baseline and Insulin biopsies will be performed on contralateral legs (one biopsy each leg). This technique allows for rapid *in vivo* sampling of skeletal muscle and adipose tissue with minimal discomfort to subjects. Muscle and adipose tissue is quickly trimmed of excess connective tissue and fat, blotted with gauze to remove blood and immediately flash frozen in liquid nitrogen and subsequently stored in liquid nitrogen (-170°C) until future analysis. Immediately following the biopsy, thin adhesive strips are used to close the wound and a breathable, transparent adhesive film dressing is applied followed by a pressure bandage. The PI will be responsible for performing the muscle biopsy procedures and the study physician will provide medical coverage and subject follow-up if necessary. The PI has been involved with hundreds of clamp procedures with muscle or adipose biopsies and hundreds of additional stand-alone skeletal muscle biopsies and stable isotope infusion studies involving exercise and metabolism in human subjects. The PI has also received specialized training by the NIH in the use of stable isotope tracers for metabolic research. The PI's experience in these techniques is demonstrated in his publication record. The PI has been approved by the University of Illinois Risk Management to perform muscle biopsies for research purposes.

Follow-up phone call: A nurse or one of the investigators will call each subject within 24 hours to follow-up on how his/her biopsy is healing and to ask about any pain or discomfort that might limit his/her activity.

<u>Additional Metabolic Testing Procedures</u>

Oral Glucose Tolerance Test: An oral glucose tolerance test (OGTT) will be used to screen subjects for abnormal glucose tolerance. Following an overnight fast of ~ 10-12 hrs, a polyethylene catheter is inserted into an antecubital vein for sampling of venous blood. Baseline blood samples are obtained after which the subject will consume a solution containing exactly 75 g of anhydrous glucose within 5 minutes. Blood samples are then

collected every 30 min for 180 minutes for the determination of glucose, insulin and other metabolic analytes. WHO and American Diabetes Association criteria are used to determine the presence or absence or abnormal glucose tolerance and type 2 diabetes mellitus. Indices of whole body insulin sensitivity will be determined using the methods of Addull-Ghani and Defronzo [ref].

Dual-emission X-ray absorptiometry (DEXA): A DEXA scan will be used to determine components of body composition such as lean body mass, fat mass, and segmental adiposity such as truncal fat and visceral fat mass. Subjects will arrive at the Integrative Physiology Lab at which time anthropometric measures of height and weight will be taken using standard techniques. After calibration, the subject will then lie supine on the bed of a GE LUNAR iDXA densitometer and a whole body scan will be performed. The duration of the scan will be less than 10 min and the subject will be exposed to a radiation dose of ≤ 0.3 mrem based upon manufacturer's specifications and calculations from Standford Dosimetry, LLC RADAR Medical Procedure Radiation Dose Calculator. This amount of research protocol radiation exposure requires no review from the Radiation Safety Committee given the estimated effective equivalent dose is below 100 mrem per year, a limit set by the Nuclear Regulatory Commission for "general public" exposure. All pre-menopausal women will undergo a point-of-care pregnancy test the day of DEXA testing. In the event of a positive test, no DEXA scan will be performed and the subject will be excluded from further study participation due to the metabolic perturbations associated with pregnancy. This test can be performed during visit 1, 2, or 3 depending upon the subject's schedule.

Analytical Assessments: The assays described below will utilize the expertise of the collaborators (Dr Fantuzzi) and the Mass Spectrometry Facility located in the UIC Research Resources Center (RRC). The RRC offers services for investigators to develop analytical methods for identification of novel bioactive compounds, and for quantification of biomarkers in biological materials such as plasma, urine and tissues. The RRC is equipped with a triple quadrupole mass spectrometer (Micromass Quattro Ultima) with electrospray ionization (ESI) capability and two HPLC systems which will provide the appropriate platform for the detection and quantification of AGEs. *AGE Determination by LC-MS/MS:* AGEs in skeletal muscle will be determined by LC-MS/MS with internal standardization by stable isotope-substituted standards as described previously (30; 31). The AGEs, methylglyoxal- and glyoxal-derived hydroimidazolone (MG-H1, G-H1), carboxyethyl-lysine (CEL), and carboxymethyl-lysine (CML) will be quantified. *Immunoblotting Studies:* Skeletal muscle homogenates separated by SDS-PAGE and protein expression/phosphorylation will be analyzed by Western blot as described (32). *Immunoprecipitation Studies:* Skeletal muscle homogenates will be precipitated (IP) with selective anti-RAGE antibodies overnight at 4°C and subsequently immunoblotted with anti-PKCα, anti-Src, anti-RAGE, anti-NFKB, and anti-IRS1 to investigate the association of the RAGE signaling complex with insulin receptor signaling proteins. Dr Fantuzzi has expertise with these methods in her Inflammation Lab at UIC***.***

Statistical Analysis. All data will be probed for the assumptions of normality by the Kolmogorov-Smirnov test and if necessary, the data will be transformed to achieve a normal distribution. Non-parametric tests (Wilcoxon-Mann-Whitney test) will be applied if transformation fails to normalize the data. For Specific Aim #1, between-group (control vs. diabetic) comparisons for AGEs and clamp derived glucose disposal rate (GDR) will be analyzed using a two-tailed, independent means t-test. Methods of collinear analysis (variance inflation factors and condition indices) will be used to establish variable independence. The significance of the correlations between variables meeting the criteria for independence and the measured responses of interest will be assessed using stepwise (forward selection with replacement) and backward regression techniques. For Specific Aim #2, differences in protein expression and activation between groups (control vs. diabetic) for each trial (basal and insulin-stimulation) of the euglycemic-hyperinsulinemic clamp will be determined via two-way (group x trial) repeated measures ANOVA. Bonferroni post hoc tests will be applied to significant group x trial interactions. Baseline values for each variable will be compared between groups using paired t-tests. Statistical significance will be accepted when $P<0.05$. Analyses will be carried out using StatView for Windows 5.0.1 (SAS Institute, NC). These proposed analyses will also be supported by the CCTS CORE services*.*

Justification of Sample Size and Power Analysis. Currently, only 3 investigations have examined AGEs in human skeletal muscle and no cross-sectional data exist on skeletal muscle AGEs or AGE/RAGE signaling between diabetic and control subjects. Thus, calculations to derive the appropriate sample size in order to detect a statistically significant effect were applied to variables associated with our primary outcome measures. These estimates were based on published data performed by the Applicant utilizing a similar study design that is being proposed in this investigation (8). Haus et al. previously demonstrated that the AGE, pentosidine, was

significantly greater in older individuals (78y; 15.9±4.5 mmol/mol collagen) compared to young (25y; 5.2±1.3 mmol/mol collagen) (8); and the effect size from this investigation was determined to be 3.23 (G*Power version 3.0.10, Kiel, Germany). An *a priori* power calculation was performed to determine the computed required sample size given: type 1 error rate of 5%, type 2 error rate of 5%, effect size of 3.23, and the statistical test selected was a two-tailed, independent means (2-sample) t-test. The resulting sample size for each group was determined to be n = 4, for a total sample size of n= 8, where actual power = 0.965. Based upon these calculations, the proposed sample size of 10 subjects for each group is adequate to detect statistical significance and account for inter-subject variation.

Table 1. Subject Commitment

**Request/Task**	**Location**	**Visit #**	**~ Time Required**	**Contact Method**
Initial Telephone Screening	Home/other	--	15 min	Telephone
Pre-Study Diet counseling	Home/other	--	15 min	Telephone or email
Full Medical Screening: Exam, Baseline blood draw, EKG, OGTT, Questionnaires	CRC	Visit 1	4 hrs	Personal
DEXA	DHSP	Visit 1 or 2 or 3*	30 min	Personal
Questionnaires (diet and activity recall)	Home	--	30 min	n/a
Metabolic Meal	AHSB	Visit 2	1 hr	Personal
Morning Urine Collection and documentation	Home	--	5 min	n/a
Indirect Calorimetry	CRC	Visit 3	30 min	Personal
Hyperinsulinemic-Euglycemic Clamp, tracer infusion, muscle biopsy, indirect calorimetry	CRC	Visit 3	6 hrs	Personal
**TOTAL**	**--**	**3 visits**	**~ 14 hrs**	**--**

***** Only one DEXA scan will be performed.

Known Discomforts and Risks

Venipuncture/Catheter Placement: The risks of drawing blood from a vein includes discomfort at the site of the needle stick, possible bruising and swelling around the site of the needle stick, rarely an infection, and uncommonly feeling faint from the procedure.

Pregnant women, fertile females/males: There may be unforeseen risks to an unborn child associated with some of the study testing. Pregnancy tests will be performed on all women of child-bearing potential before each DEXA scan.

EKG: Men may need to have a small amount of chest hair shaved off for the ECG pads to stick correctly. A small amount of adhesive might remain on the skin when the pads are removed or the adhesive might pull on hair when removed.

Questionnaires: There is the potential risk of loss of confidentiality. Every effort will be made to keep information confidential, however, this cannot be guaranteed.

Radiation Exposure DEXA scans: One of the risks associated with radiation exposure is cancer. The natural incidence of fatal cancer in the U.S. is about 1 chance in 5. Everyday radiation exposure from natural occurring background radiation (sun, radon exposure in the home) is approximately 100 mrem per year. In this research study, the subject will receive a whole body DEXA scan. The radiation dose of one wholebody DEXA scan is approximately 0.3 mrem. This amount of radiation is very low as to make an accurate risk estimate meaningless.

Study Diet: It may be burdensome for subjects to adhere to a special diet for a prolonged period of time.

Urine Collection: There are no risks to collecting urine samples. However, some people may find it uncomfortable or embarrassing to collect samples.

Indirect Calorimetry: For some people, the plastic canopy over their heads makes them feel claustrophobic or anxious. This feeling is temporary and will go away when the canopy is removed. The canopy is "see through" and does not restrict movement.

Insulin Clamp Study: There is a risk that the subject may react to the infusion of glucose or insulin. These reactions could include low blood sugar, an increase in blood pressure, flushing and/or sweating.

Heated Hand Box: There is a slight risk of skin discomfort or burn. If the temperature is too warm it can be reduced to improve comfort.

Isotope Tracer Study: The isotopes used in this study are non-radioactive tracers are present in the body naturally. They have been approved for research purposes by the US Food and Drug Administration (FDA) and used in the research setting for many years by researchers studying metabolism without adverse effects.

Muscle/adipose Biopsy: Subjects may feel pain, cramping, or bleeding where the sample is taken. Infection is very rare as aseptic procedures are used and the needle used is sterile. It is very rare, but there is a risk of allergic reaction to the lidocaine that is used to numb the subject's skin. This information will be collected in the initial telephone screening.

Oral Glucose Tolerance Test: Reaction to the ingestion of glucose. These reactions could include nausea, low blood sugar, an increase in blood pressure, flushing and/or sweating.

Unforeseeable risks: There may be risks or side effects related to the study that are unknown at this time.

Managing Adverse Events: The PI will monitor safety and review data collection on an ongoing basis. In addition, every 2 months, Dr. Haus will meet with the study monitior, Drs Unterman, MD at UIC for their review of safety and data information while the study is in the data collection phase involving human subjects. This frequency was selected given the human data collection period of this pilot study is expected to be 12 months in duration.

In the situation of an anticipated or unanticipated Adverse Event (AE), a standard IRB Adverse Event Report Form will be sent to the CRC and IRB in a timely manner. This report will include a full description of the event, including the relationship of the AE as not related, possibly related, or definitely related to the test procedure.

When reporting an AE, a common grading scale will be used:
0 – "No adverse event or within normal limits or not clinically significant"
1 – "Mild AE, did not require treatment"
2 – "Moderate AE, resolved with treatment"
3 – "Severe AE, resulted in inability to carry on normal activities and required professional medical attention"
4 – "Life threatening or disabling AE"
5 – "Fatal AE"

Data Management: The PI will examine safety and review all data on an ongoing basis. Source data will collected in data reporting forms of both electronic and written form. The PI will review these forms and data at the completion of each subject visit to ensure both accuracy and completeness. All electronic data will be secured on the PIs passwork protected computer until it can be moved to a secure UIC data server for long-term storage. Hard copy data reporting forms will be stored in a locked file cabinet until it can be scanned to

electronic copy and archived onto the UIC data server. Once files are converted the original hard copy will be kept until the study is closed, at which htime the documents will be destroyed. In the event that a discrepency or error is found in the source data, the PI will determine if the data can be collected again/procedure repeated in a subsequent study visit or alternative visit pending this will not disrupt the integrity of the data. Should this occur, the event will be documented and reported to the IRB. In addition, Every 2 months, Dr. Haus will meet with Dr Unterman, MD at UIC for their review of the data.

REFERENCES

1. Sell DR, Monnier VM: Structure elucidation of a senescence cross-link from human extracellular matrix. Implication of pentoses in the aging process. *J Biol Chem* 264:21597-21602, 1989
2. Bierhaus A, Nawroth PP: Multiple levels of regulation determine the role of the receptor for AGE (RAGE) as common soil in inflammation, immune responses and diabetes mellitus and its complications. *Diabetologia* 52:2251-2263, 2009
3. Cassese A, Esposito I, Fiory F, Barbagallo AP, Paturzo F, Mirra P, Ulianich L, Giacco F, Iadicicco C, Lombardi A, Oriente F, Van Obberghen E, Beguinot F, Formisano P, Miele C: In skeletal muscle advanced glycation end products (AGEs) inhibit insulin action and induce the formation of multimolecular complexes including the receptor for AGEs. *J Biol Chem* 283:36088-36099, 2008
4. Intensive diabetes management: implications of the DCCT and UKPDS. *Diabetes Educ* 28:735-740, 2002
5. Diabetes Control and Complications Trial (DCCT). Update. DCCT Research Group. *Diabetes Care* 13:427-433, 1990
6. Effect of intensive diabetes management on macrovascular events and risk factors in the Diabetes Control and Complications Trial. *Am J Cardiol* 75:894-903, 1995
7. Sakata N, Meng J, Jimi S, Takebayashi S: Nonenzymatic glycation and extractability of collage in human atherosclerotic plaques. *Atherosclerosis* 116:63-75, 1995
8. Haus JM, Carrithers JA, Trappe SW, Trappe TA: Collagen, cross-linking, and advanced glycation end products in aging human skeletal muscle. *J Appl Physiol* 103:2068-2076, 2007
9. Makita Z, Vlassara H, Cerami A, Bucala R: Immunochemical detection of advanced glycosylation end products in vivo. *J Biol Chem* 267:5133-5138, 1992
10. Sajithlal GB, Chithra P, Chandrakasan G: Advanced glycation end products induce crosslinking of collagen in vitro. *Biochim Biophys Acta* 1407:215-224, 1998
11. Schleicher ED, Wagner E, Nerlich AG: Increased accumulation of the glycoxidation product N(epsilon)-(carboxymethyl)lysine in human tissues in diabetes and aging. *J Clin Invest* 99:457-468, 1997
12. Meerwaldt R, Graaff R, Oomen PH, Links TP, Jager JJ, Alderson NL, Thorpe SR, Baynes JW, Gans RO, Smit AJ: Simple non-invasive assessment of advanced glycation endproduct accumulation. *Diabetologia* 47:1324-1330, 2004
13. Sell DR, Lapolla A, Odetti P, Fogarty J, Monnier VM: Pentosidine formation in skin correlates with severity of complications in individuals with long-standing IDDM. *Diabetes* 41:1286-1292, 1992
14. Meerwaldt R, Links T, Zeebregts C, Tio R, Hillebrands JL, Smit A: The clinical relevance of assessing advanced glycation endproducts accumulation in diabetes. *Cardiovasc Diabetol* 7:29, 2008
15. Takahashi M, Hoshino H, Kushida K, Inoue T: Direct measurement of crosslinks, pyridinoline, deoxypyridinoline, and pentosidine, in the hydrolysate of tissues using high-performance liquid chromatography. *Anal Biochem* 232:158-162, 1995
16. Ruster M, Franke S, Spath M, Pongratz DE, Stein G, Hein GE: Detection of elevated N epsilon-carboxymethyllysine levels in muscular tissue and in serum of patients with fibromyalgia. *Scand J Rheumatol* 34:460-463, 2005
17. Alt N, Carson JA, Alderson NL, Wang Y, Nagai R, Henle T, Thorpe SR, Baynes JW: Chemical modification of muscle protein in diabetes. *Arch Biochem Biophys* 425:200-206, 2004
18. Brown SM, Smith DM, Alt N, Thorpe SR, Baynes JW: Tissue-specific variation in glycation of proteins in diabetes: evidence for a functional role of amadoriase enzymes. *Ann N Y Acad Sci* 1043:817-823, 2005

19. Thornalley PJ, Battah S, Ahmed N, Karachalias N, Agalou S, Babaei-Jadidi R, Dawnay A: Quantitative screening of advanced glycation endproducts in cellular and extracellular proteins by tandem mass spectrometry. *Biochem J* 375:581-592, 2003
20. de la Maza MP, Uribarri J, Olivares D, Hirsch S, Leiva L, Barrera G, Bunout D: Weight increase is associated with skeletal muscle immunostaining for advanced glycation end products, receptor for advanced glycation end products, and oxidation injury. *Rejuvenation Res* 11:1041-1048, 2008
21. DeFronzo RA, Tobin JD, Andres R: Glucose clamp technique: a method for quantifying insulin secretion and resistance. *Am J Physiol* 237:E214-223, 1979
22. Kirwan JP, del Aguila LF, Hernandez JM, Williamson DL, O'Gorman DJ, Lewis R, Krishnan RK: Regular exercise enhances insulin activation of IRS-1 associated PI3-kinase in human skeletal muscle. *J Appl Physiol* 88:797-803, 2000
23. McQuire EAH, Helderman JH, Tobin JD, Andres R, Berman M: Effects of arterial versus venous sampling on analysis of glucose kinetics in man. *J Appl Physiol* 41:565-573, 1976
24. Kirwan JP, Haugel-de Mouzon S, Lepercq J, Challier J-C, Huston-Presley L, Friedman JE, Kalhan SC, Catalano PM: TNF-a is a predictor of insulin resistance in human pregnancy. *Diabetes* 51:2207-2213, 2002
25. Bergstrom J: Muscle electrolytes in man. *Scand. J. Clin. Lab Invest. Suppl.* 68:1-110, 1962
26. Kirwan JP, del Aguila LF, Hernandez JM, Williamson DL, O'Gorman DJ, Lewis R, Krishnan RK: Regular exercise enhances insulin activation of IRS-1-associated PI3-kinase in human skeletal muscle. *J Appl Physiol* 88:797-803, 2000
27. Haus JM, Carrithers JA, Carroll CC, Tesch PA, Trappe TA: Contractile and connective tissue protein content of human skeletal muscle: effects of 35 and 90 days of simulated microgravity and exercise countermeasures. *Am J Physiol Regul Integr Comp Physiol* 293:R1722-1727, 2007
28. Lemoine JK, Haus JM, Trappe SW, Trappe TA: Muscle proteins during 60-day bedrest in women: impact of exercise or nutrition. *Muscle Nerve* 39:463-471, 2009
29. Burd NA, Dickinson JM, Lemoine JK, Carroll CC, Sullivan BE, Haus JM, Jemiolo B, Trappe SW, Hughes GM, Sanders CE, Jr., Trappe TA: Effect of a cyclooxygenase-2 inhibitor on postexercise muscle protein synthesis in humans. *Am J Physiol Endocrinol Metab*, 2009
30. Ahmed N, Argirov OK, Minhas HS, Cordeiro CA, Thornalley PJ: Assay of advanced glycation endproducts (AGEs): surveying AGEs by chromatographic assay with derivatization by 6-aminoquinolyl-N-hydroxysuccinimidyl-carbamate and application to Nepsilon-carboxymethyl-lysine- and Nepsilon-(1-carboxyethyl)lysine-modified albumin. *Biochem J* 364:1-14, 2002
31. Ahmed N, Thornalley PJ: Quantitative screening of protein biomarkers of early glycation, advanced glycation, oxidation and nitrosation in cellular and extracellular proteins by tandem mass spectrometry multiple reaction monitoring. *Biochem Soc Trans* 31:1417-1422, 2003
32. del Aguila LF, Krishnan RK, Ulbrecht JS, Farrell PA, Correll PH, Lang CH, Zierath JR, Kirwan JP: Muscle damage impairs insulin stimulation of IRS-1, PI3-kinase, and Akt-kinase in human skeletal muscle. *Am J Physiol* 279:E206-212, 2000
33. Andrews NC, Faller DV: A rapid micropreparation technique for extraction of DNA-binding proteins from limiting numbers of mammalian cells. *Nucleic Acids Res* 19:2499, 1991
