# Supplementary material for: Plasma FGF21 concentrations are regulated by glucose independently of insulin and GLP-1 in lean, healthy humans
Source: PeerJ. 2022 Jan 19;10:e12755. doi: 10.7717/peerj.12755 (PMC8783558; doi:10.7717/peerj.12755)
Supplement: Supplemental Information 4 [file peerj-10-12755-s004.pdf]

Forsøgsprotokol

Projekttitel:

Effekten af interval-gang hos personer med diabetes type 2, insulin-resistens eller metabolisk syndrom.

Formål:

At undersøge effekten af interval-gang hos personer med diabetes type 2, insulin-resistens eller metabolisk syndrom.

Baggrund:

For nyligt har japansk gruppe beskrevet en ny, struktureret måde at træne på. Denne kaldes ”Interval-gang” og går kort fortalt ud på at gå skiftevis 3 minutter i moderat tempo (ca. 40 % af den maksimale iltoptagelse (VO_2-max)) og 3 minutter i hurtigt tempo (mindst 70 % af VO_2-max), begge dele gentaget minimum 5 gange hver - i alt minimum 30 minutter. Den japanske gruppe beskrev effekten af denne træning udført mindst 4 dage om ugen i minimum 5 måneder (1).
For at stimulere deltagerne til denne form for gang har japanerne udviklet en avanceret skridttæller (JD Mate), der kan indstilles til hvert tredje minut at give en bip-lyd, som tegn til at man skal skifte tempo. Tælleren kan lagre forskellige data, såvel antal skridt, hastigheden samt den tilbagelagte distance.
Studiet har, indenfor et bredt udsnit af klassiske sygdomsassocierede parametre, vist særdeles lovende resultater. Der er vist markant, signifikant og positiv effekt på muskelstyrke i benene, blodtryk, blodsukker og taljemål. Herudover øgede interval-gang VO_2-max i modsætning til, hvad man fandt for gang med moderat og konstant tempo. Interessant er, at deltager-compliance hvad angik træningen, viste sig at være særdeles stor, i og med at ca. 95 % af de inkluderede deltagere gennemførte træningsprogrammet.

Evidensen for effekten af træning på sundhed er stor. Én af de store befolkningsundersøgelser, der har haft til formål at vurdere betydningen af fysisk aktivitet for helbredet, er udført af Dr. Ralph Paffenbarger på ca. 17.000 mænd, der havde været studerende på Harvard Universitet i USA. De blev derefter fulgt i mange år. Mændene blev udspurgt om deres fysiske aktivitetsvaner. Her fandt man, at personer, der var moderat fysisk aktive i 3-4 timer pr. uge havde en reduceret risiko for død (30-40 %) i forhold til individer, der ikke var aktive (2). Et andet amerikansk studie omfattede 70.000 midaldrende kvinder. Alle kvinderne blev spurgt, hvor mange timer de gik om ugen og blev derefter fulgt i 4 år. Undersøgelsen viste en tæt sammenhæng mellem den tid kvinderne brugte på at gå og risikoen for at få diagnosticeret hjertesygdom i observationsperioden. Jo flere timer kvinden brugte på moderat fysisk aktivitet, desto mindre var risikoen for udvikling af hjerte-kar-sygdomme. Den gruppe, der gik mellem 2,5 og 5 timer pr. uge, havde en reduceret risiko for hjerte-kar-sygdomme på 30 % (3).

Det japanske studie blev udført på raske forsøgspersoner. Der findes udmærket dokumentation for at der er en dosisafhængig effekt af fysisk aktivitet, når det gælder at nedsætte risikoen for type 2

diabetes – jo mere motion, jo større beskyttelse (4), og der er tale om en effekt, der er uafhængig af kropsvægt (5). Omvendt er der i flere studier fundet en klar sammenhæng mellem fysisk inaktivitet og forekomsten af type 2 diabetes hos både mænd og kvinder (6-9).

Hvorvidt de beskrevne effekter også gælder for folk der allerede har livsstilssygdomme såsom diabetes type 2, prædiabetiske tilstande og metabolisk syndrom synes knapt så velbeskrevet. Et studie viser, at personer med type 2 diabetes kan øge deres kondital alene ved at øge antallet af daglige skridt (10). I studiet indgik 29 overvægtige mænd med type 2 diabetes og en gennemsnitsalder på 57 år, som ved lodtrækning blev inddelt i to grupper: 1) en fysisk aktivitets gruppe og 2) en kontrolgruppe. I fysisk aktivitetsgruppen fik deltagerne 1,5 times konditionstræning to gange om ugen i 2 uger. De tabte sig 2,7 % i vægt, deres kondital steg med 10 %, deres daglige antal skridt steg og deres gennemsnitlige blodsukkerniveau faldt. Den maksimale iltoptagelse, også kaldet konditallet, var stærkt korreleret til antal daglige skridt både før og efter træningsperioden.

Et nyligt, endnu ikke publiceret, studie udført bl.a. af dr. med. Allan Vaag og professor, dr. med. Niels Secher (deltagere i indeværende projekt), har vist, at den cerebrale perfusion og iltekstraktion, der hos raske personer øges under fysisk arbejde, i modsætning hertil mindskes hos folk med type 2 diabetes. En væsentlig del af forklaringen på dette menes at være det faktum at folk med type 2 diabetes ikke er i stand til at øge deres cardiac output under fysisk aktivitet. Hvorvidt dette kan ændres ved fysisk træning af type 2 diabetikere er uvist.

5-aminoimidazole-4-carboxamide riboside (AICAR) er kendt som en potent aktivator af 5′-AMP-activated protein kinase (AMPK), og infusion af AICAR er vist at kunne øge den AMPK-medierede glukoseoptagelse (12,13). Hvorvidt træning, der også er kendt som en AMPK aktivator, kan modulere den AICAR-betingede effekt er ukendt, og endvidere er forskellen mellem raske og personer med type 2 diabetes også uafklaret.

Inkretin-hormonerne Glucagon-like Peptide 1 (GLP-1) og Glucose-dependent insulinotrophic polypeptide (GIP) er kendt som ”insulinotrope” hormoner, hvilket vil sige at de øger insulin-udskillelsen fra pancreas. I de senere år har der været en stigende opmærsomhed rettet mod de ekstra-pancreatiske effekter af inkretin-hormonerne. I vores laboratorium har vi udført et mindre celleeksperiment der tyder på at GLP-1 samt GIP i sig selv medfører øget glukoseoptagelse i humane muskelceller (upublicerede data). For at undersøge denne direkte effekt af inkretin-hormonerne in vivo kan den såkaldte ”pancreatiske clamp” anvendes (14). I denne infunderes enten GLP-1 eller GIP samtidig med at Somatostatin benyttes til at modvirke den insulinotrope effekt af inkretin-hormonerne. Der infunderes tillige en konstant mængde insulin sv.t. basalkoncentrationen, væksthormon sv.t. basalkoncentrationen, samt glucagon sv.t. basalkoncentrationen (Figur 1). Glukose infunderes for at holde blodsukker-koncentrationen konstant.

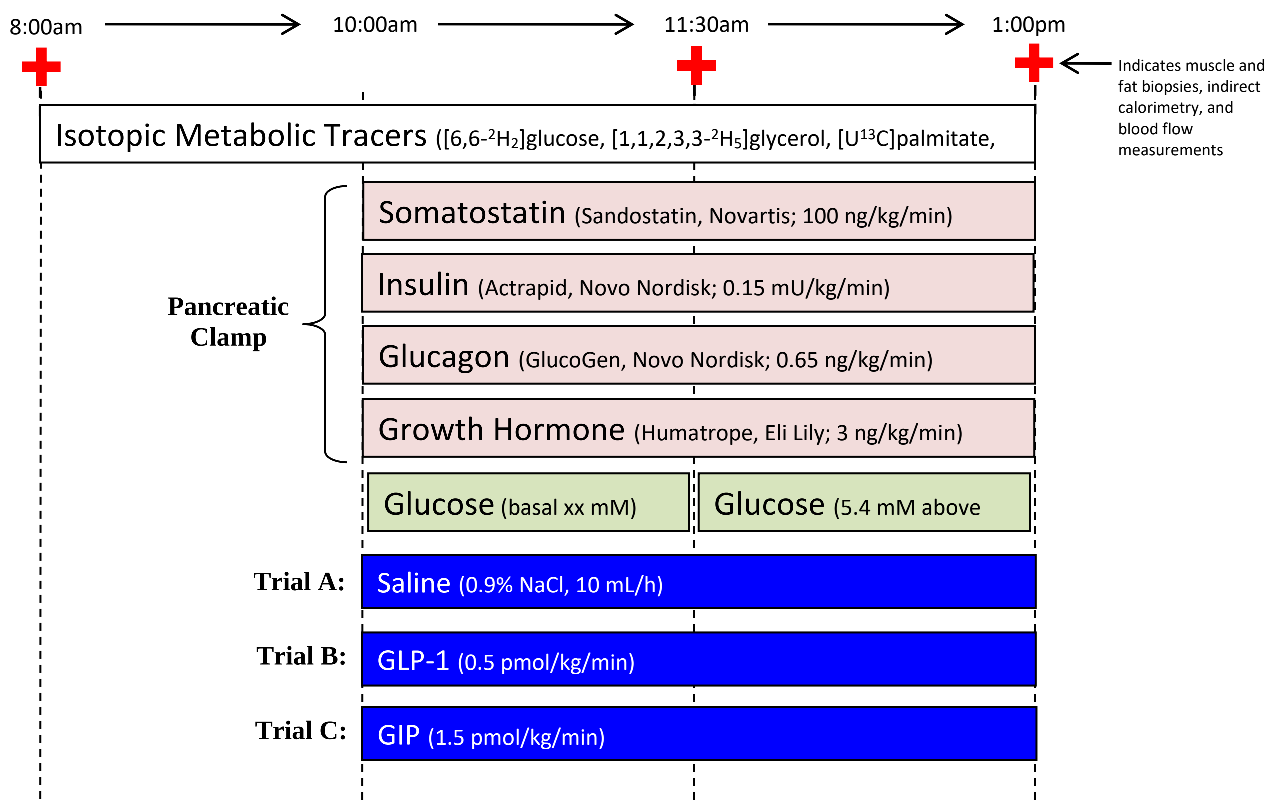

Med indeværende studie ønsker vi derfor at undersøge effekten af interval-gang hos personer med diabetes type 2, insulin-resistens eller metabolisk syndrom.
Primære endpoints vil være ”klassiske” diabetes- og sundhedsassocierede effektparametre (insulinfølsomhed, maksimal iltoptagelse, vægt, BMI, blodtryk, taljemål, hoftemål, kropskomposition, blodsukker, lipidstatus, hjerte-pumpefunktion, m.v.), mens mere grundlæggende effektparametre (ben-muskelstyrke, hjerte-pumpe-funktion, inflammatoriske mediatorer, insulin-signalering, kognitive tests, hjernens blod-flow og metaboliske aktivitet, m.v.) vil være sekundære endpoints.

Perspektivering:

Projektet, som der her søges tilladelse til, vil blive evalueret og revideret med henblik på at udvide projektet til skønsmæssigt 5.000 personer med nydiagnosticeret type 2 diabetes. Der pågår i Danmark aktuelt initiativer med henblik på at udforme en landsdækkende database af nydiagnosticerede patienter med type 2 diabetes (Danish Diabetes Type 2 Study, (DD2)). Mulige aktører er foruden et bredt udsnit af danske endokrinologer, epidemiologer og statistikere, det Strategiske Forskningsråd, Sundhedsstyrelsen og Ministeriet for Forebyggelse og Sundhed. Formålet med sidstnævnte projekt er på alle måder at optimere behandlingen af nydiagnosticerede type 2 diabetikere, med henblik på at disse undgår alvorlige følgesygdomme. Vi mener, det er væsentligt, at et landsdækkende initiativ rettet mod én af de mest betydningsfulde livsstilssygdomme, omfatter fysisk aktivitet som intervention.
I DD2 er det hensigten, at alle Danmarks 98 kommuner blive inviteret til at deltage som ”Fysisk Aktivitets Kommune” Vi skønner, at 40 kommuner vil melde sig som aktive. Blandt disse vælges

10 repræsentative kommuner, hvor alle nydiagnosticerede patienter tilbydes inklusion i interval-gangs-projektet.
Efter 5 år vurderes ”hårde endepunkter” i form af iskæmisk hjertesygdom, blodpropper i hjerte eller hjerne, samt mortalitet. Da ambitionen er at samtlige patienter i DK er inkluderet i en database, kan patienterne i de ikke inkluderede kommuner fungere som kontrol-kommuner.
Det vil forud for DD2 være utrolig vigtigt, at der foreligger valide data om effekten af interval-gang i en dansk befolkning med type 2 diabetes eller forstadier hertil, hvorfor vi betragter projektet dækket af indeværende ansøgning som absolut nødvendigt at gennemføre.

<u>Forsøgsdeltagere:</u>

Inklusionskriterier:
100 mænd eller kvinder ≥ 18 år med et eller flere af følgende karakteristika:

- Type 2 diabetes (2-hour OGTT > 11.1 mmol/l)
- Insulinresistens (Impaired glukose tolerance (IGT) ; 2-hour OGTT > 7.8 mmol/l)
- Metabolisk syndrom, forstået som faste-blodsukker > 5,6 mmol/l samt én eller flere af følgende risikofaktorer:
  - Hvile BT systolisk > 140 mmHg
  - Livvidde (mænd) > 102 cm
  - Livvidde (kvinder) > 88 cm
  - BMI > 25 kg/m^2
  - Hyperkolesterolæmi (det aktuelle cut-off afhænger af om der er andre risici eller dispositioner)

- Forsøgspersonens accept af at pausere perorale antidiabetika, antihypertensiva samt statiner og lignende stoffer 1 uge inden forsøgsdagene.

Eksklusionskriterier:
- Graviditet
- Sygdom der kontraindicerer fysisk aktivitet (11)
- Insulin afhængig diabetes mellitus
- BMI < 19 eller > 40 kg/m^2
- Thyreoideaforstyrrelser
- Ikke stabil vægt (> 2 kg vægttab eller –øgning gennem sidste 6 måneder)
- Fysisk aktivitet (≥ 30 min/dag, ≥ 3 dage/uge)

Endvidere vil der i forsøget indgå 15-20 raske alders- og BMI-matchede kontrol-personer..

<u>Rekruttering, information samt indhentning af samtykke:</u>

Forsøgsdeltagere rekrutteres ved direkte kontakt, annoncering på ”www.forsøgsperson.dk” samt opslag i dagspressen. De kontakter en af projektdeltagerne telefonisk og informeres om projektet. Såfremt der ikke ved den telefoniske kontakt identificeres eksklusionskriterier, aftales fremmøde i afdelingen og forsøgsdeltageren oplyses om muligheden for at have bisidder med ved fremmødet. Forud herfor fremsendes skriftlig patientinformation inkl. samtykkeerklæring.

Ved fremmøde i afdelingen afholdes informationssamtale af en de videnskabelige deltagere i projektet. Samtalen afholdes i lukket lokale hvor kun videnskabelig deltager, forsøgsperson samt evt. bisidder vil være til stede.
Efter informationssamtalen vil forsøgspersonen blive opfordret til (igen) at gennemlæse den skriftlige deltagerinformation samt at overveje evt. deltagelse i forsøget. Forsøgspersonen oplyses om muligheden for at kontakte videnskabelig deltager telefonisk, i tilfælde af opklarende spørgsmål. Der aftales telefonisk kontakt indenfor ca. 1 uge (og evt. før i tilfælde af opklarende spørgsmål), mhp. om forsøgspersonen ønsker at indgå i forsøget. Såfremt dette er tilfældet medbringes underskrevet samtykkeerklæring til screenings-undersøgelsen.

Screening
Lægelig undersøgelse, anamnese, biokemisk screening for evt. anden sygdom samt oral glukose tolerance test (OGTT).

Styrkeberegning:
Tidligere undersøgelser har vist store forbedringer i en af vore primære effektparametre (insulinfølsomhed) efter livsstils-interventioner. Baseret på en estimeret effektstørrelse på 0,85, en type 1 fejlprocent på 5, og en type 2 fejlprocent på 20, er det beregnet, at mindst 12 personer i hver gruppe skal fuldføre studiet for at der kan påvises statistisk signifikante resultater. Således finder vi det ønskede antal forsøgspersoner tilstrækkeligt og passende.

Metoder:

For samtlige forsøgspersoner påtænkes følgende basale undersøgelser udført før og efter en 6-måneders træningsperiode. Undersøgelserne vil blive splittet op i 2 på hinanden følgende dage:

Dag -3:
3 dages registrering af
- Fysisk aktivitet ved hjælp af Actiheart accelerometer og hjertefrekvens-måler
- Fødeindtag
- Blodglukose (Guardian real-time continuous glucose monitoring system)

samt
- 1 døgns urin-opsamling til analyse af inflammatoriske markører.

Dag 1:
- Vejning, BMI, bestemmelse af hvilepuls, blodtryk, livvidde, hoftevidde
- Bestemmelse af basal metabolisme vha. indirekte kalorimetri
- Udfyldelse af spørgeskema vedrørende træning
- Kognitiv test (computer-baseret (CPT II))

- OGTT eller Mixed Meal tolerance test (som OGTT men med måltid bestående af 55% kulhydrat, 30% fedt og 15% protein, med energiindhold på 10 kcal/kg)
- Blodprøvetagning med undersøgelse af lipidstatus (kolesterol samt triglycerid), inflammatoriske parametre (TNF, IL-6, CRP, m.v.), hæmatologiske parametre (Hb, Hct,), faste-blodsukker, HbA1C, pro-BNP
- Direkte bestemt maksimal iltoptagelse (VO_2-max test)

Dag 2:
- Styrkemåling af benmuskler
- Helkrops DXA-skanning (mhp. at evaluere kropskomposition samt knoglemineraltæthed)
- MR-skanning mhp. kvantificering af femoral muskelmasse samt abdominal fedme

For 60 forsøgspersoner, vil der herudover blive foretaget følgende udvidede undersøgelser, udført over 2 på hinanden følgende dage:

Dag 3a:
- Clamp-undersøgelse (hyperinsulinæmisk (insulininfusionsrate = 40 $mU/m^2/min$), eu- og hyperglykæmisk (glukosekoncentration = 5-12 mM)) med tracere i form af stabile glukose-isotoper
- Glucagon like peptide 1 (GLP-1) infusion intravenøst (GLP-1 infusionsrate = 1 pmol/kg/min) eller Glucose-dependent insulinotrophic polypeptide (GIP) infusion intravenøst (GIP infusionsrate = 20 ng/kg/min)
- IntraLipid™ infusion intravenøst (FFA-koncentration = 1 mM)
- Arginin hydroclorid bolus injektion (mhp. bestemmelse af β-celle reservekapacitet)
- Biopsitagning (af muskel- samt fedtvæv)
- AICAR infusion (Infusionsrate 10 mg/kg/time)

<u>eller</u>

Dag 3b:
- Pancreatisk Clamp-undersøgelse (eu- og hyperglykæmisk (glukosekoncentration = 5-12 mM)), med indgift af somatostatin (Sandostatin (Novartis): 100 ng/kg/min), insulin (Actrapid (Novo Nordisk): 0,15 mU/kg/min), Glucagon (GlucaGen (Novo Nordisk): 0,65 ng/kg/min) og væksthormon (Humatrope (Eli Lily): 3 mg/kg/min) samt med tracere i form af stabile glucose-, bikarbonat- samt lipidisotoper.
- GLP-1 infusion (infusionsrate = 0,5 pmol/kg/min) eller GIP infusion (infusionsrate = 1,5 pmol/kg/min) eller NaCl-infusion (kontrol)
- Biopsitagning (af muskel- samt fedtvæv)

Dag 4:
- MR-skanning (mhp. bestemmelse af cerebralt blod-flow, cerebral metabolisk aktivitet, bestemmelse af størrelse af cerebrale centre)
- Transtorakal EKKO-kardiografi mhp. uddrivningsfraktions-bestemmelse (LVEF)
- Transkranial doppler undersøgelse mhp. at bestemme hjernens blodflow
- Anlæggelse af CVK samt arterie-kanyle i a. radialis / a. femoralis mhp. at bestemme cerebral oxygenering

Forud for forsøgsdagene vil personerne i 3 dage skulle registrere deres fødeindtag, hvilket vil blive analyseret.
Forsøgspersonerne vil blive instrueret i at undlade fysisk aktivitet i 48 timer forud for forsøgsdagene.
På forsøgsdagen skal forsøgspersonerne møde fastende op (min. 6 timers faste). Efter blodprøvetagning vil de kunne spise frit.

Vedr. biologisk materiale:

I forbindelse med projektet planlægges udtaget biologisk materiale i form af blod, urin samt fedt- og muskelbiopsier. Dette vil blive benyttet til at vurdere biokemiske såvel som strukturelle ændringer hos de involverede forsøgspersoner.
Der udtages ca. 200 ml. blod pr. forsøgsdag for forsøgspersoner der gennemgår basale undersøgelser og ca. 500 ml. blod pr. forsøgsdag for forsøgspersoner der gennemgår udvidede undersøgelser. I alt udtages altså mellem ca. 400 og 1000 ml. blod under hele forsøget.
For forsøgspersoner der gennemgår udvidede undersøgelser udtages 2-4 fedtbiopsier fra abdomen og 2-4 muskelbiopsier fra m. quadriceps femoris, vastus lateralis, pr. forsøgsdag. Hver biopsi udgør ca. 100-200 mg.
Det biologiske materiale vil under forsøgets fulde længde (ca. 3-5 år) blive opbevaret i en forskningsbiobank, og vil efterfølgende overgå i Center for Inflammation og Metabolisme's samlede biobank. Såfremt evt. senere studier ønsker at gøre brug af det biologiske materiale vil dette kun finde sted efter godkendelse fra etisk komite.
Det biologiske materiale forlader ikke Danmark.

Træningsperioden:

Forsøgspersoner instrueres initialt i anvendelse af JD Mate skridttæller indtil de er sikre i anvendelsen.
Herefter udfører forsøgspersonerne træning i form af interval-gang gennem 6 måneder, mindst 30 minutter/dag, mindst 5 gange/uge.
For de 60 forsøgspersoner der undergår udvidede undersøgelser vil 20 blive randomiseret til at udføre interval-gang som ovenfor beskrevet, mens 20 vil blive randomiseret til at udføre ikke-interval-gang med samme energiforbrug som intervalgangsgruppen gennem 6 måneder, mindst 30 minutter/dag, mindst 5 gange/uge. Endelig vil 20 personer blive inkluderede som kontrolpersoner der ikke skal foretage ændringer i deres træningsmængde.
Alle forsøgspersoner møder på vores laboratorium hver 14. dag gennem hele forsøget, hvor chippen aflæses og hvor de får en papirudskrift af deres indsats. Såfremt træningsindsatsen ikke er tilfredsstillende, jf. tidligere beskrivelse, vil forsøgspersonerne blive opfordret til at øge deres træningsindsats.

Risici, bivirkninger og ubehag:

Pause af perorale antidiabetika, antihypertensiva samt statiner:
For at kunne vurdere effekten af den fysiske træning er det nødvendigt at forsøgspersonerne op til forsøgsdagene pauserer ovenstående medikamenter. Disse medikamenter tages over og virker over lang tid og det vurderes betydningsløst at pausere dem i kortere perioder.

Blodprøver:
Ved blodprøvetagning er der ubehag i form af nålestik. Blodmængden er så lille, at der ikke er symptomer ved dette.

Kontinuerlig blodglukose-monitorering:
En sensor indføres i subcutis og sidder her i 3 dage. Forsøgspersonen bærer en mindre monitor i en bæltetaske og blodglukose-værdier sendes automatisk til og lagres i denne. Sensoren indføres let og med minimalt ubehag og skønnes ikke at medføre ubehag når den sidder i subcutis.
Der er en ganske lille risiko for infektion i huden i forbindelse med proceduren.
Blodglukose-monitorering er tidligere benyttet til videnskabelige formål i vores laboratorium.

VO_2-max test:
Der foretages en direkte konditionstest, hvor forsøgspersonerne skal præstere det maksimale. Der opstår nogen grad af åndenød.

DXA-skanning, EKKO-kardiografi og transkraniel Doppler-undersøgelse:
Skønnes ikke at medføre væsentligt ubehag.

Clamp-undersøgelser:
Der anlægges venflons mhp. indgift af insulin, glukose, mv., samt til blodprøvetagning. Der vil være gener svarende til en blodprøvetagning, og der vil være risiko for dannelsen af hæmatom i forbindelse med fjernelse af venflonet.
I øvrigt vurderes der ikke at være risici forbundet med ovenstående for forsøgspersonen.
Forsøgspersonen vil være nøje overvåget og forbydes at sove under forsøget. Inden forsøgspersonen forlader afdelingen vil der blive serveret glukoseholdigt måltid og blodsukker vil blive kontrolleret.

GLP-1 / GIP infusion:
GLP-1 er et i kroppen naturligt forekommende hormon. Det kan medføre gastro-intestinale gener og hovedpine.
GLP-1 er tidligere benyttet til videnskabelige formål i vores laboratorium.
GIP er et naturligt forekommende hormon som kroppen secernerer efter fødeindtagelse, og som hos raske personer medfører øget insulinsekretion. GIP har tidligere været benyttede til videnskabelige formål af en af deltagerne i projektet (JJ Holst).

Arginin hydrochlorid injektion:
Arginin er en i kroppen naturligt forekommende aminosyre. Det kan medføre forbigående gastro-intestinale gener samt hovedpine.

Stabile isotoper (benyttet i clamps samt OGTT):
Der vil blive infunderet stabile sukkerisotoper mhp. bestemmelse af glukoseoptagelse og –dannelse samt lipid- og bikarbonatisotoper mhp. bestemmelse af indirekte kalorimetri og substratforbrug.

Isotoperne er ikke radioaktive, sikre at anvende, har tidligere været benyttet til videnskabelige formål i vores laboratorium, og skønnes ikke at medføre andet ubehag for forsøgspersonen end nævnt under clamp-undersøgelsen.

Biopsitagning:
Udtagningen af biopsier foretages i lokalbedøvelse. Anlæggelsen af lokalbedøvelse kan give en forbigående sviende smerte i huden. Herefter kan resten af indgrebet som regel foretages uden væsentlig ubehag eller smerte. Der er ofte lettere lokal ømhed efter biopsierne i 1-3 dage. I enkelte tilfælde kan der opstå en større blodudtrædning omkring indstiksstedet, hvilket medfører en kraftigere ømhed (”trælår”), som kan vare flere dage. For at reducere risikoen for større blodudtrædninger, bliver forsøgspersonerne opfordret til tidligst at genoptage motion 1 døgn efter den sidste biopsi.
I meget sjældne tilfælde kan en mindre nervegren i huden blive skåret over ved udtagningen af en biopsi. Dette kan give føleforstyrrelser af et hudområde nedenfor såret igennem flere måneder. Herefter forsvinder føleforstyrrelserne som oftest.

AICAR infusion:
AICAR har tidligere været infunderet i såvel raske forsøgspersoner (12) som i forsøgspersoner med type 2 diabetes (13) uden nævneværdige bivirkninger. Endvidere viser farmakokinetiske og -dynamiske undersøgelser af stoffet god tolerabilitet samt sikkerhed ved doser på 10 gange det i indeværende studie påtænkte (15).

Infusion af somatostatin-analog, glucagon samt væksthormon:
Den korte periode hvor stofferne gives betyder at risikoen for bivirkninger er begrænset: Sandostatin samt GlucaGen kan medføre gastrointestinale gener samt allergiske reaktioner. Endvidere er beskrevet hypo- og hyperglykæmi hvilket der kontrolleres for med hyppige blodsukker-målinger (hvert 5. min) samt justering af glukoseinfusions-raten. Den øvre anbefalede grænse for daglig indgift af sandostatin er 1,5 mg (www.medicin.dk), hvilket vil blive respekteret.
Humatrope kan ligeledes medføre allergiske reaktioner og er beskrevet at kunne medføre forbigående myalgier og arthralgier. Der er rapporteret tilfælde af leukæmi hos børn behandlet med Humatrope; noget der aldrig er set hos voksne (16).

MR-scanninger:
Forsøgspersoner vil inden skanning skulle udfylde et standardiseret spørgeskema, for at sikre at der ikke er nogen kontraindikationer for en MR-skanning. MR-skanninger af abdomen og ekstremiteter er tidligere benyttet til videnskabelige formål i vores laboratorium, og de cerebrale MR-skanninger vil blive udført i samarbejde med dr. med. Allan Vaag. Skanningerne skønnes ikke at medføre væsentligt ubehag for forsøgspersonen.

Anlæggelse af CVK og A-kanyle:
I lokal-bedøvelse anlægges ultralydsvejledt CVK i v. jugularis samt arteriekateter i a. radialis / a. femoralis. Anlæggelsen af lokalbedøvelse kan give en forbigående sviende smerte i huden. Herefter kan resten af indgrebet som regel foretages uden væsentlig ubehag eller smerte. Der er ofte lettere lokal ømhed hvor katetrene har været anlagt, men denne vil være forbigående. I sjældne tilfælde giver CVK-anlæggelsen anledning til pneumo- eller hæmothorax mens såvel CVK- som a-kanyle anlæggelse sjældent kan give anledning til blødning eller infektion.
Anlæggelsen af katetrene vil blive foretaget af læger med erfaring i de nødvendige teknikker. Forsøgspersonerne vil være under konstant overvågning under disse invasive procedurer.

Sikkerhedsforanstaltninger:

Forsøget indebærer ikke ekstra sikkerhedsforanstaltninger.

Forsikringsforhold:

Forsøgspersoner er dækket af hospitalets forsikring.

Etiske aspekter:

Projektet forventes i henhold til ovenstående at medføre begrænsede risici, bivirkninger og ubehag for forsøgspersonerne. Omvendt vurderes forsøget at medføre fordele for forsøgspersonerne, i og med at de vil blive introduceret til struktureret og superviseret træning i form af interval-gang.
For forskningen generelt vurderes, i henhold til perspektiveringsafsnittet, at projektet er absolut nødvendigt og på sigt med betydelig sandsynlighed vil bidrage til folkesundheden i en stor og stadig voksende gruppe af folk med livsstilssygdomme.

Anmeldelse af projektet til Datatilsynet:

Projektet vil blive anmeldt til datatilsynet.

Økonomisk støtte:

Bente Klarlund Pedersen har taget initiativ til projektet.
Projektet er støttet økonomisk af Trygfonden med i alt 985.000 kr, og endvidere finansieret af bevillinger til Center for Inflammation og Metabolisme (CIM) på i alt 650.000 kr.
Ingen af projektdeltagerne har i øvrigt økonomisk tilknytning til Trygfonden.

Fysiske rammer for udførelse af forsøget:

Epidemiklinikken og Centeret for Muskelforskning, Rigshospitalet, Tagensvej 20, afsnit 7641
Steno Diabetes Center, Niels Steensens Vej 2, 2820 Gentofte

Tidsplan:

Projektet iværksættes umiddelbart efter godkendelse af etisk komite. Den eksperimentelle del forventes at være tilendebragt i løbet af 18 mdr. Derefter pågår analysearbejde og bearbejdelse af data, samt sammenskrivning af videnskabelige artikler, i alt endnu ca. 18 mdr. Såvel positive som negative resultater vil blive offentliggjort.

Honorar:

Der ydes en godtgørelse på 3000,- kr., for forsøgspersoner der deltager i de basale undersøgelser. For deltagelse i de udvidede undersøgelser ydes en godtgørelse på yderligere 3000,- kr., altså i alt 6000,- kr. Godtgørelsen er skattepligtig. Godtgørelsen dækker påført ubehag, tabt arbejdsfortjeneste og transportudgifter. Honoraret udbetales efter forsøgspersonens udtræden af projektet. Såfremt forsøgspersonen bliver ekskluderet eller vælger at udtræde af forsøget, vil der blive udbetalt et beløb svarende til den del af studiet personen har gennemført.

Deltagere i projektet:

Kristian Karstoft, læge, klinisk assistent, Rigshospitalet, Finsencenteret, Epidemiklinikken
Thomas Solomon, cand. scient, Ph.D., post. doc., Rigshospitalet, Finsencenteret, Epidemiklinikken
Maria Pedersen, læge, stud Ph.D., Rigshospitalet, Finsencenteret, Epidemiklinikken
Rikke Krogh-Madsen, læge, Ph.D., post. doc., Rigshospitalet, Finsencenteret, Epidemiklinikken
Allan Vaag, overlæge, dr. med., Steno Diabetes Center, Novo Nordisk A/S
Niels Secher, professor, overlæge, dr. med., Rigshospitalet, anæstesiologisk klinik
Jens Juul Holst, professor, dr. med., Københavns Universitet, Fysiologisk Institut
Bente Klarlund Pedersen, professor, overlæge, dr. med., Rigshospitalet, Finsencenteret, Epidemiklinikken

Referencer

1. Nemoto K, Gen-No H, Masuki S, Okazaki K, Nose H: Effects of high-intensity interval walking training on physical fitness and blood pressure in middle-aged and older people. *Mayo Clin Proc* 82:803-811, 2007

2. Paffenbarger RS Jr., Kampert JB, Lee IM, Hyde RT, Leung RW, Wing AL: Changes in physical activity and other lifeway patterns influencing longevity. *Med Sci Sports Exerc* 26:857-865, 1994

3. Manson JE, Greenland P, LaCroix AZ, Stefanick ML, Mouton CP, Oberman A, Perri MG, Sheps DS, Pettinger MB, Siscovick DS: Walking compared with vigorous exercise for the prevention of cardiovascular events in women. *N Engl J Med* 347:716-725, 2002

4. Hu FB, Sigal RJ, Rich-Edwards JW, Colditz GA, Solomon CG, Willett WC, Speizer FE, Manson JE: Walking compared with vigorous physical activity and risk of type 2 diabetes in women: a prospective study. *JAMA* 20;282:1433-1439, 1999

5. Hu G, Lindstrom J, Valle TT, Eriksson JG, Jousilahti P, Silventoinen K, Qiao Q, Tuomilehto J: Physical activity, body mass index, and risk of type 2 diabetes in patients with normal or impaired glucose regulation. *Arch Intern Med* 164:892-896, 2004

6. Carnethon MR, Gidding SS, Nehgme R, Sidney S, Jacobs DR Jr., Liu K: Cardiorespiratory fitness in young adulthood and the development of cardiovascular disease risk factors. *JAMA* 290:3092-3100, 2003

7. Manson JE, Rimm EB, Stampfer MJ, Colditz GA, Willett WC, Krolewski AS, Rosner B, Hennekens CH, Speizer FE: Physical activity and incidence of non-insulin-dependent diabetes mellitus in women. *Lancet* 338:774-778, 1991

8. Manson JE, Nathan DM, Krolewski AS, Stampfer MJ, Willett WC, Hennekens CH: A prospective study of exercise and incidence of diabetes among US male physicians. *JAMA* 268:63-67, 1992

9. Hu G, Qiao Q, Silventoinen K, Eriksson JG, Jousilahti P, Lindstrom J, Valle TT, Nissinen A, Tuomilehto J: Occupational, commuting, and leisure-time physical activity in relation to risk for Type 2 diabetes in middle-aged Finnish men and women. *Diabetologia* 46:322-329, 2003

10. Bjorgaas M, Vik JT, Saeterhaug A, Langlo L, Sakshaug T, Mohus RM, Grill V: Relationship between pedometer-registered activity, aerobic capacity and self-reported activity and fitness in patients with type 2 diabetes. *Diabetes Obes Metab* 7:737-744, 2005

11. Pedersen BK, Saltin B: Evidence for prescribing exercise as therapy in chronic disease. *Scand J Med Sci Sports* 16 Suppl 1:3-63.:3-63, 2006

12. Cuthbertson DJ, Barbraj JA, Mustard KJ, Towler MC, Green KA, Wackerhage H, Leese GP, Baar K, Thomason-Hughes M, Sutherland C, Hardie DG, Rennie MJ. 5-aminoimidazole-4-carboxamide 1-beta-D-ribofuranoside acutely stimulates skeletal muscle 2-deoxyglucose uptake in healthy men. *Diabetes.* 2007 Aug;56(8):2078-84

13. Boon H, Bosselaar M, Praet SF, Blaak EE, Saris WH, Wagenmakers AJ, McGee SL, Tack CJ, Smits P, Hargreaves M, van Loon LJ. Intravenous AICAR administration reduces hepatic glucose output and inhibits whole body lipolysis in type 2 diabetic patients. *Diabetologia.* 2008 Oct;51(10):1893-900.

14. Basu A, Charkoudian N, Schrage W, Rizza RA, Basu R, Joyner MJ. Beneficial effects of GLP-1 on endothelial function in humans: dampening by glyburide but not by glimepiride. *Am J Physiol Endocrinol Metab* 293:E1289-E1295, 2007

15. Dixon R, Gourzis J, McDermott D, Fujitaki J, Dewland P, Gruber H. AICA-riboside: safety, tolerance, and pharmacokinetics of a novel adenosine-regulating agent. *J Clin Pharmacol*. 1991 Apr;31(4):342-7.

16. Lægemiddelstyrelsens Produktresumé (http://www.produktresume.dk/docushare/dsweb/Get/Document-15982/Humatrope%2C+pulver+og+solvens+til+injektionsv%C3%A6ske%2C+opl%C3%B8sning+6+mg%2C+12+mg+og+24+mg.doc)
