# Supplementary material for: Plasma FGF21 concentrations are regulated by glucose independently of insulin and GLP-1 in lean, healthy humans
Source: PeerJ. 2022 Jan 19;10:e12755. doi: 10.7717/peerj.12755 (PMC8783558; doi:10.7717/peerj.12755)

CONSORT flow diagrams for the three independent studies:

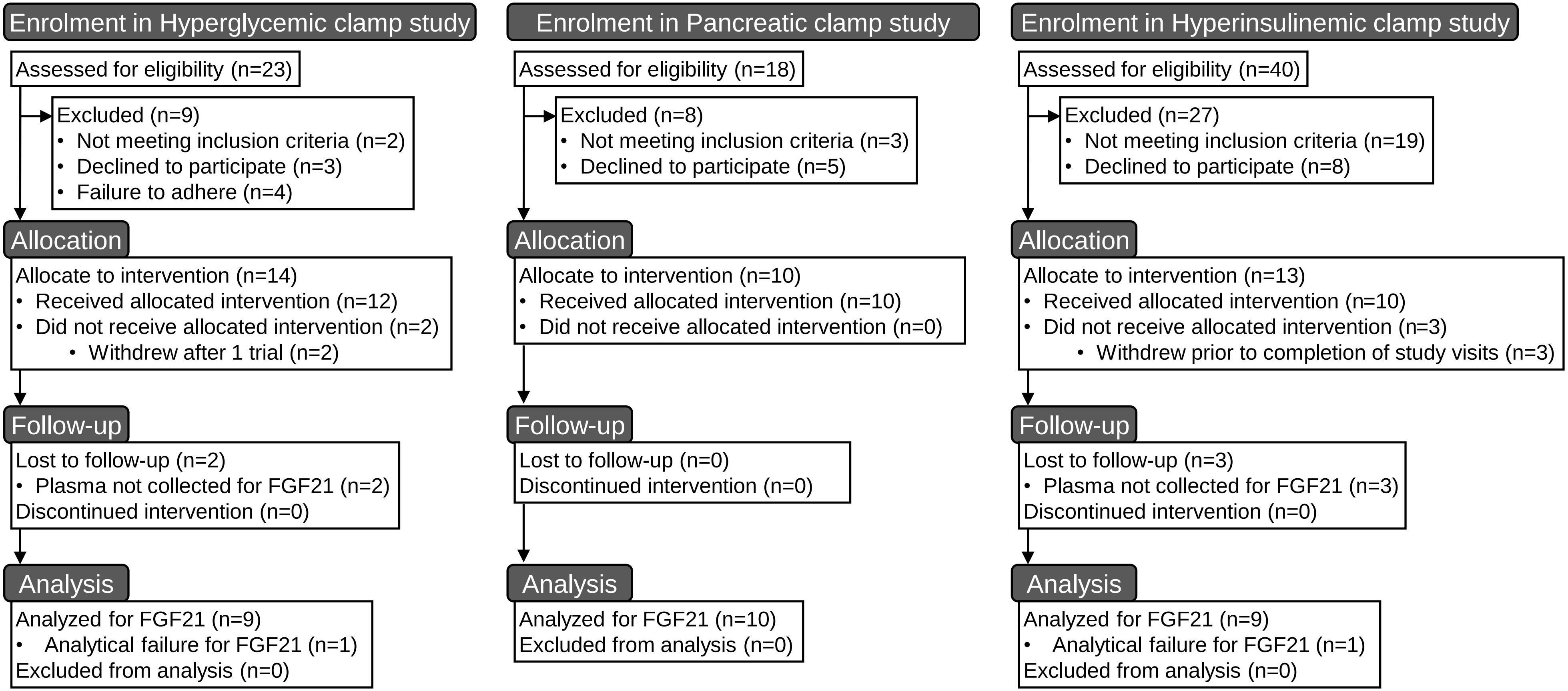

Supplement: Supplemental Information 5 [file peerj-10-12755-s005.pdf]
